# Food environment research in Canada: a rapid review of methodologies and measures deployed between 2010 and 2021

**DOI:** 10.1186/s12966-024-01558-x

**Published:** 2024-02-19

**Authors:** Caroline Vaillancourt, Mavra Ahmed, Sara Kirk, Marie-Ève Labonté, Amos Laar, Catherine L. Mah, Leia Minaker, Dana Lee Olstad, Monique Potvin Kent, Véronique Provencher, Rachel Prowse, Kim D. Raine, Ashley Schram, Daniela Zavala-Mora, Maryka Rancourt-Bouchard, Lana Vanderlee

**Affiliations:** 1 École de Nutrition, Centre de Nutrition, Santé et Société (NUTRISS), Université Laval, 2425 Rue de L’Agriculture, Québec, QC G1V 0A6 Canada; 2 Department of Nutritional Sciences, University of Toronto, 1 King’s College Circle, Toronto, ON M5S 1A8 Canada; 3 School of Health and Human Performance, Dalhousie University, 6230 South Street, Kjipuktuk (Halifax), NS B3H 4R2 Canada; 4 Department of Population, Family and Reproductive Health, School of Public Health, University of Ghana, P. O. Box LG 13, Legon, Accra Ghana; 5 School of Health Administration, Dalhousie University, 5850 College Street, Halifax, NS B3H 4R2 Canada; 6 School of Planning, University of Waterloo, 200 University Avenue West, Waterloo, ON N2L 3T1 Canada; 7 Department of Community Health Sciences, University of Calgary, 3280 Hospital Drive NW, Calgary, AB T2N 4Z6 Canada; 8 School of Epidemiology and Public Health, University of Ottawa, 600 Peter Morand Crescent, Ottawa, ON K1G 5Z3 Canada; 9 Division of Community Health and Humanities, Faculty of Medicine, Memorial University of Newfoundland, 300 Prince Philip Drive, St. John’s, NL A1B 3V6 Canada; 10 School of Public Health, University of Alberta, 11405 87 Ave Northwest, Edmonton, AB T6G 1C9 Canada; 11 School of Regulation and Global Governance (RegNet), ANU College of Asia & the Pacific, The Australian National University, 8 Fellows Road, Canberra, Australian Capital Territory 2600 Australia; 12 Science Library, Université Laval, 1045 Avenue de La Médecine, Québec, QC G1V 0A6 Canada

**Keywords:** Food environments, Monitoring, Research methodologies and measures, Food marketing, Food labelling, Food prices, Food provision, Food composition, Food retail, Food trade and investment

## Abstract

Numerous research methodologies have been used to examine food environments. Existing reviews synthesizing food environment measures have examined a limited number of domains or settings and none have specifically targeted Canada. This rapid review aimed to 1) map research methodologies and measures that have been used to assess food environments; 2) examine what food environment dimensions and equity related-factors have been assessed; and 3) identify research gaps and priorities to guide future research. A systematic search of primary articles evaluating the Canadian food environment in a real-world setting was conducted. Publications in English or French published in peer-reviewed journals between January 1 2010 and June 17 2021 and indexed in Web of Science, CAB Abstracts and Ovid MEDLINE were considered. The search strategy adapted an internationally-adopted food environment monitoring framework covering 7 domains (*Food Marketing; Labelling; Prices; Provision; Composition; Retail; and Trade and Investment*). The final sample included 220 articles. Overall, *Trade and Investment* (1%, *n* = 2), *Labelling* (7%, *n* = 15) and, to a lesser extent, *Prices* (14%, *n* = 30) were the least studied domains in Canada. Among *Provision* articles, healthcare (2%, *n* = 1) settings were underrepresented compared to school (67%, *n* = 28) and recreation and sport (24%, *n* = 10) settings, as was the food service industry (14%, *n* = 6) compared to grocery stores (86%, *n* = 36) in the *Composition* domain. The study identified a vast selection of measures employed in Canada overall and within single domains. Equity-related factors were only examined in half of articles (*n* = 108), mostly related to *Retail* (*n* = 81). A number of gaps remain that prevent a holistic and systems-level analysis of food environments in Canada. As Canada continues to implement policies to improve the quality of food environments in order to improve dietary patterns, targeted research to address identified gaps and harmonize methods across studies will help evaluate policy impact over time.

**Supplementary Information:**

The online version contains supplementary material available at 10.1186/s12966-024-01558-x.

## Introduction

Evidence has increasingly indicated that individual dietary intakes and dietary patterns are heavily shaped by people’s food environments [1–3]. Environmental factors such as food access, availability, cost, and marketing across a variety of settings can support or inhibit healthier diets at a population level [3]. Improving the quality of food environments is an important policy goal for chronic disease prevention worldwide, as policymakers and key public health figures shift focus from individual-level behaviours and ‘lifestyle’ factors to broader structural determinants of dietary intake [4, 5].

The increased interest in improving the quality of food environments has compelled governments and researchers to identify key policy and research questions related to monitoring the state of food environments as a part of dietary risk factor surveillance. For instance, in Canada, the *Healthy Eating Strategy*, announced in 2016, identified key policy recommendations regarding food labelling, composition and marketing to children to improve the health of Canadians [6]. As of 2023, the Strategy has resulted in revisions to Canada’s Food Guide [7], a ban on the use of partially hydrogenated oils in foods [8], changes to food labelling including mandatory front-of-package nutrition symbol labelling regulations in 2022 that will be fully implemented by 2026 [9, 10], and updates to Canada’s sales-weighted voluntary targets for sodium reduction [11, 12]. As part of the Strategy, Health Canada has also proposed amendments to the *Food and Drug Regulations* to restrict advertising to children of foods high in sodium, sugars and saturated fat [13]. There is a need for robust and consistent measures to effectively monitor those environments as well as to evaluate the contribution of policy actions to dietary, nutrition and health outcomes [4, 14]. Research methods and outcomes currently in use to assess the quality of food environments in Canada and globally vary greatly, even when measuring the same characteristic or outcome, thereby limiting opportunities to compare or benchmark across jurisdictions and settings. Consequently, it is important to better understand the landscape of existing food environment measures. Lastly, changes to food environments are one potential avenue to address inequities in healthy eating [15]. The importance of reducing inequities in dietary intake warrants an in-depth look at how equity-related factors are being studied in food environment research to better understand how food environments may generate and/or exacerbate existing inequities.

Several previous systematic reviews have examined food environment research, many of which evaluated the association between specific food environment measures and health- or diet-related outcomes [16–20], and several of which have examined food environment metrics and methods used to assess those environments [21–26]. Depending on each review’s objectives, these were restricted to a single domain, such as food marketing environments [26], examined limited settings [23], focused on specific countries or types of countries [22, 25] or reviewed research related to specific population groups [21]. The first major review on food environment measures by McKinnon et al. that specifically aimed to compile the literature on the measurement approaches used broadly, neither covered dimensions such as web-, television-, and other media-based marketing nor quantitatively assessed how frequently outcomes were assessed in the literature [24]. A subsequent systematic review, building on this previous review, included solely food environment measures in schools, restaurants, workplaces, or stores, and excluded literature addressing food prices, a critical element of food environments [23]. Others have focused specifically on the community nutrition environment (e.g., density of food outlets) [22] or examined the methods used to study food environments specifically in low- and middle-income countries [25], or assessed the associations between food environment and dietary, nutrition and health outcomes without explicitly compiling methods and measurements used [16–19]. To our knowledge, there is no existing assessment across multiple food environment domains that provide a holistic view of the food environment research that has been recently conducted in Canada. This is important, as it is increasingly acknowledged that food environments are inextricably linked and operate at a systems level [1]. For researchers aiming to assess the food environment at the macro-level, understanding research gaps across domains and identifying links between methodologies across policy areas may facilitate improved monitoring and evaluation. It may also support more in-depth analyses within policy domains or areas. Finally, taking such a holistic review approach collates data and information into one location, which may support more effective policy development and implementation by knowledge users. The key nutrition policy actions currently being undertaken by the federal government with the *Healthy Eating Strategy* [6] and additional novel policies including sugary drink taxes at a provincial level [27–30] warrant extensive evaluation. A comprehensive understanding of the food environment research and methods being used in Canada, including documenting existing knowledge and existing gaps, is necessary and timely to continue to inform and advance food policy in Canada. Therefore, the objectives of this rapid review were to 1) map research methodologies and measures that have been used to evaluate food environments in Canada; 2) examine what food environment dimensions and equity-related factors have been assessed; and 3) identify research gaps and priorities to guide future research.

## Methods

### Rapid review methodology

A rapid review approach was selected given that we aimed to timeously identify research gaps in order to set priorities and guide future research on the measurement of food environments in Canada. Synthesizing the strength of the evidence or providing recommendations regarding the most appropriate methodologies were beyond the scope of the current review. Rapid reviews are recognized by the World Health Organization (WHO) to be of utmost importance to informing health policy and systems and have proven to be useful to provide relevant evidence in a shortened timeframe and cost-effective manner, as well as to identify areas where future primary research should be targeted [31]. The methodology for this study was adapted from articles providing methodological guidance for systematic and scoping reviews [32–34]. Overall, a systematic approach was used but with several accelerated approaches, including a less extensive search (only three databases were used), abstract and full-text screening and data extraction were completed by a single reviewer, and no bias or quality appraisal was conducted [35, 36]. Reporting follows the Preferred Reporting Items for Systematic Reviews and Meta-Analyses (PRISMA) checklist for items applicable to the present work (Additional file 1) [37].

### Literature search

A systematic search was conducted to capture peer-reviewed literature evaluating the Canadian food environment using Web of Science, CAB Abstracts and Ovid MEDLINE databases. The searches were conducted between June 15 and 17, 2021. In this review, food environments are defined as the collective *physical*, *economic*, *policy* and *sociocultural* surroundings, opportunities and conditions that influence people’s dietary patterns and nutritional status, and we employ the conceptual framework developed by the International Network for Food and Obesity Research, Monitoring and Action Support (INFORMAS) that has identified 7 key domains to include in comprehensive food environment monitoring: Food Composition; Food Labelling; Food Provision; Food Marketing; Food Retail; Food Prices; and Food Trade and Investment (Table 1) [4]. Hereafter, these domains are referred to, respectively, Composition; Labelling; Provision; Marketing; Retail; Prices; and Trade and Investment. They represent characteristics of food environments as they relate to obesity and diet-related non-communicable diseases that are impacted by policies and actions of public and private sector organizations in regard to create healthy food environments [4]. The search strategy was developed with a librarian using the INFORMAS monitoring framework and used text words and relevant indexing terms to capture concepts related to 1) food; 2) food environment; 3) evaluation, assessment or monitoring; and 4) Canada (and each province and territory). Common terminology associated with each of the 7 domains was also included. The search strategy developed for Ovid MEDLINE and adaptations made for other databases are presented in Additional file 2. The results were uploaded into EndNote software, duplicates were removed, and the remaining citations were transferred to Covidence.

**Table 1:** Description of the food environment characteristics monitored for each domain within the adapted INFORMAS monitoring framework used in this study [4]

**Food retail**	Aims to monitor the geographic patterning of retail food outlets at the community level and the availability, placement and promotion of foods at the consumer level (in-store and in restaurants)
**Food marketing**	Aims to monitor the extent and nature of food marketing that population groups (especially children) are exposed to across various media and settings
**Food composition**	Aims to monitor the nutrient composition (e.g., sodium, saturated fat, sugar, and energy levels) and the nutritional quality of the food supply in food retail (e.g., supermarkets) or services settings (e.g., quick-service restaurants)
**Food provision**	Aims to monitor the foods provided or sold in key public sector settings (e.g., schools, hospitals and recreation and sport settings) and compile information on existing food or nutrition policies and/or programs and quality of foods provided or sold relative to existing national or sub-national nutrition standards or voluntary guidelines
**Food prices**	Aims to monitor the price and affordability of foods and diets
**Food labelling**	Aims to monitor the nature and extent of health-related labelling components on food packaging
**Food trade and investment**	Aims to monitor the risks to food environments within trade and investment agreements by examining tariffs applied to 'healthy' food vs. 'less healthy' food categories; import and export volumes of 'healthy'/'less healthy' foods; import quotas and commitments regarding agricultural domestic support and foreign investment related to the food processing, retailing, and advertising industry

### Study selection

**Fig. 1:**
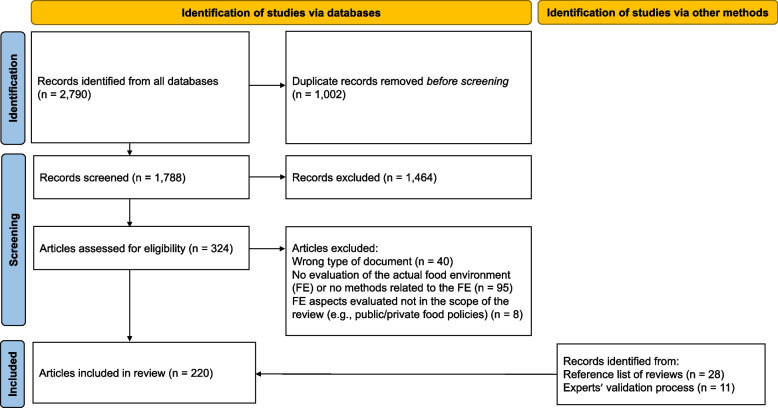
Flow diagram for study selection process

Peer-reviewed articles were considered for inclusion in the review if they met the following criteria: 1) published in English or French; 2) published in peer-reviewed journals between January 2010 and June 2021; 3) assessed the overall ‘food environment’ or at least 1 specific domain of the food environment; and 4) performed in a real-world setting (experimental studies or studies using solely prediction or simulation models were excluded). A cut-off of January 2010 was used to capture the most recent methods that are being employed in research and monitoring efforts, and to provide up-to-date literature on the topic that could be used to inform current policy decisions. Only primary research articles were included. Reviews were not included but were retained to hand search references lists for additional relevant publications. Studies assessing the food environment subjectively, for example through opinions or perceptions of participants or stakeholders only were excluded. Researchers with expertise in measuring food environments were solicited to validate the final list of articles selected for inclusion in the review and suggested any missing articles. The study selection process is summarized in Fig. 1.

To ensure consistency, a screening tool was developed, pilot-tested and used to guide the screening process and ensure consistency (Additional file 3). Two independent reviewers screened titles and abstracts of the first 168 records, in 4 distinct rounds (respectively 33, 30, 30 and 75 records). Inter-reviewer agreement (i.e., titles and abstracts classified the same way – retained or excluded – by both reviewers) was high, ranging between 88 and 96%. Disagreements were resolved after each round through discussion between reviewers. Following each round, the screening tool was refined as needed. Records marked as ‘unclear’ or remaining conflicts were discussed with a third researcher and criteria were clarified as needed. The remaining titles and abstracts (*n* = 1620) were screened by a single reviewer. Thirty-seven full texts were screened by 2 independent reviewers. Inter-reviewer agreement was acceptable (75%). Full-text screening for the remaining articles (*n* = 287) was completed by 1 reviewer.

### Data collection and extraction

A data extraction form was created and pilot-tested with an article related to outcomes for each food environment domain (i.e., 7 articles) and iterative revisions were made to ensure consistency in the data extraction process. The form was also reviewed by food environment researchers and refined according to their suggestions. Data extraction was completed by a single reviewer. The following data were extracted: year of publication; food environment domains; jurisdiction level; study settings; methodologies; outcomes or indicators used to assess the food environment; and equity factors (described in detail below) accounted for in the evaluation of food environments.

### Data coding, analysis and synthesis

Data coding was performed according to the INFORMAS monitoring framework and the description of each food environment domain (Table 1). Each publication was first coded by domain that the study examined. If multiple domains were assessed in a single publication, it was assigned to several domains. For example, an article related to the Retail domain using a tool evaluating various components such as food availability and food prices was coded only in the Retail domain if a global score for all those food environment variables was reported, or in both the Retail and Prices domains if sub-scores for each component were reported. The jurisdictional level (i.e., national, provincial or territorial, regional and municipal) was attributed to a publication according to the level at which the food environment was evaluated and referred to the geographically bounded area that the author referenced. Therefore, an article aiming to analyze the nutrient composition of the Canadian food supply was coded as ‘national jurisdiction’ even if foods and beverages analyzed were sampled in 3 out of the 13 provinces and territories in Canada. Regional jurisdictions captured locations smaller than a province or territory but larger than a single municipality (e.g., Southern Ontario, Avalon Peninsula in Newfoundland and Labrador). The types of settings were informed by the INFORMAS monitoring framework [4], and additional categories were developed as needed. For the Retail domain more specifically, the *consumer retail food environment* referred to environment in stores or restaurants (e.g., food availability, product placement), whereas the *community retail food environment* referred to type, location and accessibility of food outlets in the community [38].

To summarize and harmonize the methods and outcomes, categories were coded loosely based on existing literature [22–24, 39–41]. Methodological attributes of the evaluation were recorded across 3 components: 1) data sources; 2) data collection methods; and 3) methodological details related to the analysis (e.g., types of food outlets exposure measures for Retail, systems used to classify claims for Labelling).

The codes created for the equity-related factors (e.g., gender, age, socioeconomic status) were based on previous literature [42] and factors captured all elements of the PROGRESS (referring to Place of residence; Race/ethnicity/culture/language; Occupation; Gender/sex; Religion; Education; Socioeconomic status; and Social capital) framework proposed by the Cochrane Equity Methods Group to be considered when addressing equity in interventions or systematic reviews [43]. Publications were coded as accounting for equity-related factors if 1) analyses examined the impact of equity-related factors on the outcomes; and/or 2) if equity factors were taken into account in the sampling strategy to ensure representation in the study of specific characteristics related to equity (e.g., women, low-income neighborhoods). For studies that were conducted specifically in an understudied setting such as in a rural region, an underprivileged setting (e.g., low-to-medium income neighborhoods), among vulnerable communities (e.g., Cree women, northern communities) or when equity was considered in the study design (e.g., adaptation of an assessment tool to include foods representing ethno-cultural diversity), the articles were also considered as accounting for equity-related factors.

## Results

### Rapid review study selection process

**Fig. 2:**
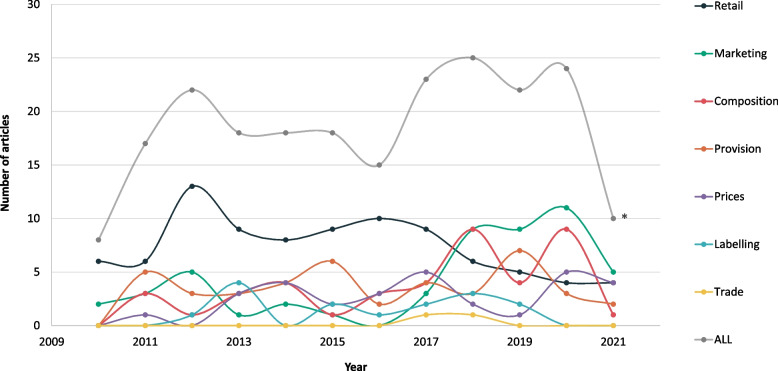
Number of articles assessing the food environment in Canada published by year for each domain and overall. *2021 data represent only half a year

A total of 220 articles assessing food environments in Canada published from 2010–2021 met inclusion criteria and were included in the review (Fig. 1). The number of publications per year varied, with an average of 18 articles per year (Fig. 2). Among articles included, 1 was published in French [44] and the remaining were English publications. The curves illustrating the annual number of articles published in each food environment domain show a decrease in publications related to Retail since 2017, and an increase in publications related to Marketing.

Some methodological articles that appeared in the search were excluded as only indicators to measure food environments or development of monitoring tools or frameworks were discussed, without being accompanied by an assessment of the actual food environments [45–48].

### Food environment domains

Of the 220 articles, the Retail food environment was most frequently studied (40%, *n* = 89), followed by the Marketing (23%, *n* = 51), Composition (19%, *n* = 42), Provision (19%, *n* = 42) and Prices (14%, *n* = 30) domains (see Tables 3, 4, 5, 6, 7 and 8 for more detail). The Labelling (7%, *n* = 15) and Trade and Investment domains (1%, *n* = 2) were addressed to a lesser extent (Table 2).

**Table 2:** Number of articles that assessed more than 1 food environment domains

Food environment domains	n	Reference numbers
*2 domains*		
Retail, Prices	14	[49–62]
Retail, Provision	6	[63–68]
Prices, Provision	1	[69]
Composition, Prices	4	[70–73]
Composition, Marketing	4	[74–77]
Composition, Labelling	5	[78–82]
Provision, Marketing	2	[83, 84]
Marketing, Labelling	1	[85]
Marketing, Prices	1	[86]
Marketing, Retail	1	[87]
*3 domains*		
Composition, Labelling, Marketing	2	[88, 89]
Prices, Marketing, Retail	3	[90–92]
Labelling, Composition, Prices	1	[93]
**Total**	**45**	

Tables 3, 4, 5, 6, 7 and 8 summarize the jurisdiction levels in which the studies were conducted, the study settings, the methods used and the outcomes that have been assessed for each food environment domain. As more than 1 domain, setting, method and/or outcome may have been identified from a single publication, the counts in those tables should only be interpreted as a proxy for relative popularity, rather than an actual frequency of use.

**Table 3:** Characteristics of articles associated with the food retail domain (*n* = 89)

**Variables**		**n**	**%**	**References numbers**
**Jurisdiction level**	National	10	11	[64, 68, 94–101]
	Provincial/territorial	13	15	[52, 57, 65, 66, 90–92, 102–107]
	Regional	13	15	[50, 51, 53, 54, 59, 67, 108–114]
	Municipal^a^	53	60	[40, 41, 49, 55, 56, 58, 60–63, 87, 115–156]
**Setting**	Community retail food environment	72	81	[40, 41, 50, 53–55, 58, 63–66, 68, 94–100, 102–106, 108–113, 115–156]
	Consumer retail food environment	24	27	[49–57, 59–62, 67, 87, 90–92, 101, 107, 114, 116, 117, 132]
**Methods**	**Data sources to identify food stores (for all settings)**			
	Commercial data	52	58	[40, 50, 56, 58, 60, 64–66, 68, 94–100, 102, 104–106, 108, 112, 115, 117, 119–129, 131, 134, 136–138, 142, 143, 145, 147, 148, 150–156]
	Administrative data	35	39	[41, 49, 50, 53–55, 59, 62, 63, 103, 109–111, 113–118, 122, 123, 125, 127, 130–135, 139–141, 145, 146, 150]
	Ground-truthing	10	11	[50, 53, 54, 56, 61, 62, 112, 122, 139, 144]
	Other (e.g., academic database, farmers’ market associations)	10	11	[49, 63, 115, 122, 123, 125, 127, 145, 149]
	Not specified or applicable	9	10	[51, 52, 57, 67, 87, 90, 92, 101, 107]
	**Analysis of food outlets ‘exposure’ or accessibility** (for community retail food environment, *n* = 72)			
	Place-based measure	65	90	[40, 41, 53–55, 58, 63–66, 68, 95–100, 102–106, 109, 111, 112, 115–128, 130–144, 146–156]
	People-based measure (i.e., use of individual’s mobility data)	4	6	[41, 110, 129, 155]
	Use of purposely designed buffers	52	72	[53, 54, 58, 63–66, 68, 97–100, 102–106, 109, 111, 112, 115–118, 121, 125, 127, 128, 130–132, 134–141, 143, 144, 146–156]
	**Data collection methods** (for consumer retail food environment, *n* = 24)			
	Observational audit (in-store)	23	96	[49–57, 59–62, 67, 87, 90, 101, 107, 114, 116, 117, 132]
	Other	3	13	[92, 116, 132]
**Outcomes**	Community retail food environment (*n* = 72)			
	Density of food outlets	55	76	[40, 41, 53, 58, 63, 68, 94–100, 102–106, 109, 111, 112, 115, 117–123, 125, 127, 129–143, 146–148, 150–155]
	Proximity to food outlets	19	26	[53, 58, 104, 106, 113, 115, 117, 124–126, 131–133, 136–138, 145, 146, 156]
	Food outlets availability (presence)	7	10	[55, 65, 66, 108, 144, 147, 149]
	Accessibility to food outlets (other than distance-related)	5	7	[115, 117, 126, 128, 145]
	Other	5	7	[50, 53, 110, 125, 147]
	Consumer retail food environment (*n* = 24)			
	Food availability (type of products)	11	46	[49–55, 59, 61, 62, 67]
	Food prominence (shelf-space)	8	33	[49, 51, 53, 56, 62, 87, 90, 101]
	Food variety (number of distinct products)	7	29	[56, 57, 61, 62, 91, 92, 114]
	Food placement (product location)	4	17	[56, 87, 101, 114]
	Food quality (organoleptic properties)	3	13	[56, 62, 114]
	Food quality (‘healthiness’)	3	13	[53, 54, 60]
	Overall setting ‘healthiness’	3	13	[51, 116, 132]
	Other	4	17	[53, 60, 61, 107]

^a^Also include 2 articles in which the studies was conducted on a university campus [61] or in a city’s neighborhood [62]

**Table 4:** Characteristics of articles associated with the food marketing domain (*n* = 51)

**Variables**		**n**	**%**	**References numbers**
**Jurisdiction level**	National	27	53	[74–77, 85, 86, 88, 89, 157–175]
	Provincial/territorial	11	22	[83, 84, 90–92, 176–181]
	Regional	1	2	[182]
	Municipal	12	24	[87, 183–193]
**Setting**	Television	18	35	[160, 161, 165, 166, 168, 169, 171–173, 176–178, 182, 184–186, 188, 189]
	Digital	11	22	[157, 162, 163, 167, 169–173, 175, 190]
	Food packaging	10	20	[74–77, 88, 89, 158, 159, 164, 183]
	Stores or restaurants	9	18	[86, 87, 90–92, 169, 171–173]
	Print	6	12	[169, 171–174, 182]
	School settings	5	10	[83, 84, 172, 173, 191]
	Recreational sports settings	5	10	[172, 173, 179–181]
	Outdoor (billboards, signs, vehicles with product or brand marketing)	4	8	[171–173, 192]
	Events (sport or concert)	4	8	[171–173, 182]
	Giveaways, samples, or special offers	4	8	[169, 171–173]
	Sponsorship	4	8	[169, 171, 173, 187]
	Movie theatres	3	6	[172, 173, 193]
	Radio	3	6	[172, 173, 182]
**Methods**	**Data collection methods (all settings)**			
	In-store	13	25	[74, 76, 77, 85–90, 158, 159, 164, 183]
	Commercial database	10	20	[91, 92, 163, 168, 170, 184–186, 188, 189]
	Questionnaire	8	16	[83, 84, 169, 171–173, 182, 190]
	Online audit (for food packaging and digital media)	6	12	[75, 157, 162, 167, 175, 187]
	Observational audit	5	10	[179–181, 191, 193]
	TV station recording	5	10	[160, 161, 176–178]
	TV viewing diary	3	6	[176–178]
	Governmental database	2	4	[165, 166]
	Other (e.g., print media, ground-truthing)	3	6	[166, 174, 192]
**Outcomes**	Exposure to marketing	40	78	[74, 76, 83, 85–87, 91, 92, 160–163, 165–182, 184–193]
	Types of foods marketed^a^	28	53	[74, 76, 77, 89, 158–161, 163–165, 167, 168, 170, 175, 176, 178, 184–193]
	Quality (‘healthiness’) of foods marketed	21	41	[75–77, 86, 88, 158, 160, 161, 163, 164, 170, 177–181, 185, 189, 190, 192, 193]
	Power of marketing	20	39	[85, 86, 90, 157, 159–162, 167, 175–177, 179–181, 184–186, 190, 191]
	Nutrient content of food marketed	13	25	[74, 75, 85, 88, 89, 163, 167, 170, 177, 178, 183–185]
	Food companies that advertised^b^	11	22	[86, 163, 166, 167, 170, 175, 183, 184, 187, 190, 193]
	Presence of child-appealing marketing	4	8	[74, 89, 162, 167]
	Location of marketing (e.g., at entryway, at checkout)	3	6	[86, 90, 193]
	Serving size of foods marketed	2	4	[88, 89]
	Other (e.g., social media use)	5	10	[83, 84, 162, 181, 190]

^a^Includes publications in which the identification of types of foods marketed is an explicit outcome as well as publications in which results are reported by food categories

^b^Includes publications in which the identification of food companies is an explicit outcome as well as publications in which results are reported by food companies

**Table 5:** Characteristics of articles associated with the food composition domain (*n* = 42)

**Variables**		**n**	**%**	**References numbers**
**Jurisdiction level**	National	37	88	[73–82, 88, 89, 93, 194–217]
	Provincial/territorial	2	5	[70, 218]
	Regional	2	5	[71, 72]
	Municipal	1	2	[219]
**Setting**	Grocery stores	36	86	[70–82, 88, 89, 93, 194–210, 217–219]
	Food service industry (e.g., restaurants)	6	14	[211–216]
	Other (e.g., drugstores, foods from the land for studies in Indigenous communities)	2	5	[72, 202]
**Methods**	**Method of nutritional content analysis**			
	Research database	39	93	[71, 73–82, 88, 89, 93, 194–216, 218, 219]
	Government database	3	7	[71, 72, 210]
	Non-governmental (or commercial) database	2	5	[70, 217]
	Laboratory analysis	2	5	[204, 205]
	Other	2	5	[72, 210]
**Outcomes**	Nutrient content	37	88	[70–75, 78–82, 88, 89, 93, 194–196, 198–205, 208–219]
	Quality (‘healthiness’) of foods	13	31	[71, 75–78, 88, 89, 197, 201, 206, 208–210]
	Presence of a specific nutrient or ingredient^a^	7	17	[78, 93, 199, 202, 207, 215, 217]
	Package or serving size	4	10	[75, 80, 200, 213]
	Product sub-categories (type of ready-to-eat cereals)	1	2	[70]

^a^Includes, for example, the frequency of various type of sugars, the use of artificial sweeteners, the presence of (partially) hydrogenated oils or whole grains

**Table 6:** Characteristics of articles associated with the food provision domain (*n* = 42)

**Variables**		**n**	**%**	**References numbers**
**Jurisdiction level**	National	3	7	[64, 68, 220]
	Provincial/territorial	30	71	[65, 66, 83, 84, 221–246]
	Regional	4	10	[44, 67, 247, 248]
	Municipal	5	12	[63, 69, 249–251]
**Setting**	School settings	28	67	[63–66, 68, 69, 83, 84, 220–230, 233–236, 245, 247–250]
	Recreation and sport settings	10	24	[231, 232, 237–244]
	Childcare settings	3	7	[44, 246, 251]
	Healthcare settings	1	2	[67]
**Methods**	**Data collection methods**			
	Observational audit	25	60	[65, 66, 68, 69, 221, 225, 226, 228–233, 237, 239–245, 247–249, 251]
	Questionnaire (self-reported)	16	38	[44, 64, 83, 84, 220, 222–224, 226, 227, 231, 234, 235, 237, 243, 248]
	Interviews with key stakeholders	11	26	[44, 63, 68, 225, 227–230, 238, 239, 242]
	Document review	5	12	[236, 237, 239, 242, 251]
	Weighing of school meals	2	5	[246, 250]
	Digital photography of school meals	2	5	[246, 249]
	Ethnographic methods	1	2	[67]
**Outcomes**	Food availability	26	62	[44, 64–69, 83, 220, 222–226, 228–230, 235, 236, 240, 241, 243, 246, 247, 249, 251]
	Healthy eating initiatives or practices^a^	18	43	[44, 63, 64, 68, 84, 220, 222, 223, 227, 228, 230, 231, 237, 238, 241, 243, 248, 251]
	Adherence to provincial nutrition policy/guidelines	16	38	[221, 224, 225, 227, 234, 235, 237–239, 241–245, 247, 248]
	Food quality (‘healthiness’)	15	36	[227, 228, 231–233, 235–237, 239–243, 247, 249]
	Nutrient content of foods/meals	8	19	[69, 239, 240, 242, 244, 246, 249, 250]
	Access to food facilities (e.g., water fountains, cafeteria, tuck shop or vending machines)	6	14	[64–66, 68, 247, 251]
	Overall setting ‘healthiness’^b^	4	10	[239, 241–243]

^a^Includes, for example, formal school initiatives to promote healthy eating (other than provincial guidelines or policy-related initiatives), such as cooking classes, presence of a food committee, nutrition training for staff, etc.

^b^Represented by a global score from a survey tool (e.g., Nutrition Environment Measures Survey for restaurants for restaurant settings (NEMS-R)) assessing multiples dimensions or outcomes

**Table 7:** Characteristics of articles associated with the food prices domain (*n* = 30)

**Variables**		**n**	**%**	**References numbers**
**Jurisdiction level**	National	4	13	[73, 86, 93, 252]
	Provincial/territorial	8	27	[52, 57, 70, 90–92, 253, 254]
	Regional	10	33	[50, 51, 53, 54, 59, 71, 72, 255–257]
	Municipal^a^	8	27	[49, 55, 56, 58, 60–62, 69]
**Setting**	Grocery retailers	24	80	[49–59, 62, 70, 71, 73, 86, 90–93, 252–255]
	Convenience stores	6	20	[53, 54, 58, 90–92]
	Restaurants	3	10	[53, 58, 60]
	Other (e.g., drugstores, natural stores, farmer's market, discount stores)	11	37	[51, 59, 61, 62, 69, 72, 90–92, 256, 257]
**Methods**	**Data collection methods**			
	In-store (observational audit, survey or census)	25	83	[49–62, 69, 71, 73, 86, 90, 93, 252–254, 256]
	Online audit	3	10	[60, 252, 255]
	Commercial database	3	10	[70, 91, 92]
	Other (ethnographic methods, publicly available information such as reports)	3	10	[72, 255, 257]
**Outcomes**	Food prices of specific foods or food categories	23	77	[49, 52, 53, 55–62, 69–73, 90–93, 252, 256, 257]
	Food pairs comparison^b^	9	30	[50, 51, 53–55, 58, 61, 62, 86]
	Diet cost	4	13	[53, 71, 252, 255]
	Diet affordability	2	7	[253, 254]
	Other (e.g., frequency of price promotion, value of a food hamper)	4	13	[52, 91, 92, 256]
	**Price metric category used** (among publications reporting on price of specific foods or food groups; *n* = 18)			
	Per unit of weight (price per kilogram, price per 100 g and price per pound)	10	56	[49, 50, 56, 57, 71–73, 86, 256, 257]
	Per unit or piece	6	33	[49, 56, 61, 70, 93, 256]
	Per serving	5	28	[50, 56, 70, 91, 92]
	Per unit of energy (price per 100 kilocalories or price per 1000 kilocalories)	2	11	[50, 71]
	Other or undefined	2	11	[51, 69]

^a^Also include 2 articles in which the studies were conducted on a university campus [61] or in a city’s neighborhood [62]

^b^Refers to the comparison of the cost of pairs of similar items with a difference in nutrient content (e.g., whole wheat vs white pasta) or of group of ‘healthier’ items and their ‘less healthy’ counterparts

**Table 8:** Characteristics of articles associated with the food labelling domain (*n* = 15)

**Variables**		**n**	**%**	**References numbers**
**Jurisdiction level**	National	15	100	[78–82, 85, 88, 89, 93, 258–263]
**Setting**	Grocery stores (prepackaged foods)	15	100	[78–82, 85, 88, 89, 93, 258–263]
**Methods**	**Data collection methods**			
	In-store	15	100	[78–82, 85, 88, 89, 93, 258–263]
	**System used to classify food labels/claims**			
	Canadian regulations	5	33	[79, 81, 258, 261, 263]
	INFORMAS taxonomy^a^	1	7	[262]
	Institute of Medicine definition of FOPL^b^	2	13	[260, 263]
	Developed by the research team (for unregulated symbols)	2	13	[79, 261]
	Not mentioned/Not applicable	8	53	[78, 80, 82, 85, 88, 89, 93, 259]
**Outcomes**	Types of claims or symbols	14	93	[78–82, 85, 89, 93, 258–263]
	Types of foods with claims	12	80	[78, 79, 81, 82, 85, 93, 258–263]
	Quality (‘healthiness’) of foods with claims	6	40	[78, 79, 85, 258, 259, 262]
	Nutrient content of foods with claims	2	13	[258, 260]
	Other (e.g., food package size)	2	13	[80, 88]

^a^System proposed by INFORMAS for classifying the health-related labelling components on packaged foods and based on the Codex food labelling standards and guidelines [264]

^b^Definition used in the Institute of Medicine (IOM) Front-of-Package Label (FOPL) Committee releases Phase 1 report (2010)

### Characteristics of articles assessing the food retail domain

#### Jurisdiction and settings

Retail food environments were most frequently assessed at the municipal level (60%) and to a lesser extent at the regional (15%), provincial or territorial (15%), and national (11%) levels (Table 3). The community retail food environment was assessed in 81% of Retail articles, and the consumer retail food environment was analyzed in 27% of Retail articles (Table 3). The community retail food environment was frequently assessed within administrative units like census tracts or dissemination areas (38%, *n* = 27) [40, 41, 50, 53–55, 94–96, 108–110, 115–129], around schools (33%, *n* = 24) [63–66, 68, 97–100, 102–105, 111, 112, 130–138], around residences (36%, *n* = 26) [58, 104, 106, 113, 133, 135–137, 139–156] and around recreation centres or workplace (3%, *n* = 2) [130, 140]. The consumer retail food environment included in-store (79%, *n* = 19) [49–57, 59, 62, 67, 87, 90–92, 101, 114, 117] and restaurant settings (25%, *n* = 6) [53, 60, 61, 107, 116, 132].

#### Methods and outcomes

To identify food stores within a specific area, the most popular data sources among all Retail articles were commercial data (58%) (e.g., Enhanced Points of Interest (EPOI) database from the Desktop Mapping Technologies Inc., Yellow Pages), administrative data from jurisdictions applying to the study setting (39%) and ground-truthing (11%). In articles related to the community retail food environment (*n* = 72), analysis of food outlets ‘exposure’ or accessibility were most frequently performed with place-based measures (90%), among which 66% (*n* = 43) used fixed spatial units such as areas around schools [53, 54, 58, 63–66, 68, 97–100, 102–106, 111, 112, 127, 130–132, 134–141, 143, 144, 146–152, 154–156] or residences, or area-based anchors (45%, *n* = 29) such as census tracts [40, 41, 50, 55, 95, 96, 106, 108, 109, 115–128, 133, 139–142, 153]. Fewer publications (6%) assessed food outlets exposure through people-based measures, such as individual's Global Positioning System (GPS) trajectory data or travel survey data, which take into account the various food environments people get exposed to when they accomplish their daily routine by tracking and mapping people’s daily mobility and activities [41]. In the majority of articles related to the community retail food environment (72%), buffers were purposely designed by researchers to define the study area, either using straight line (42%, *n* = 22; i.e., radial buffer) [53, 54, 64–66, 68, 97–100, 102, 104, 105, 111, 112, 115, 117, 130, 135–137, 150] or road network (52%, *n* = 27) measures [58, 99, 100, 103, 104, 109, 116, 118, 120, 121, 128, 132, 134, 138–141, 143, 144, 146–149, 153–156]. There was no mention on the type of buffers used in 13% of publications (*n* = 7) [63, 102, 114, 125, 133, 151, 152]. Fifteen different sizes of buffers were used, ranging from 200 m to 8 kms around a specific location, with the most popular sizes being 1000 m (*n* = 26) [53, 54, 64, 65, 97–100, 104–106, 111, 112, 115, 117, 118, 125, 127, 130, 135–137, 146, 150, 154, 155], 500 m (*n* = 14) [54, 58, 63, 68, 99, 111, 128, 130, 135, 148, 149, 151, 152, 156] and 800 m (*n* = 6) [58, 102, 116, 131, 132, 143]. In 3 articles, a 10- or 15-min walking distance was also used to define network buffers [120, 139, 153]. Among articles that applied buffers, 17% (*n* = 9) used more than 1 buffer size [54, 58, 99, 104, 111, 118, 130, 132, 135]. For data collection methods in articles related to the consumer retail food environment (*n* = 24), nearly all data were collected through observational audits (96%). Other studies collected online information or used commercial databases (13%).

The community retail food environment was most frequently operationalized as the density of food outlets (76%). Among articles assessing density (*n* = 55), 85% (*n* = 47) [40, 53, 58, 63, 68, 97–100, 102–106, 109, 111, 112, 115, 117–121, 125, 127, 130–143, 146–148, 150–154] used area density measures, such as the number of food outlets within a buffer zone or within a square kilometer, 9% (*n* = 5) [94–96, 122, 123] used outlets to population ratio and 6% (*n* = 3) used density measure based on individual's mobility [41, 129, 155]. Measures of proximity, availability and accessibility (other than distance-related, such as accessibility in terms of cost of transportation, average public transit or walking travel time to food outlets, hours of operation of food outlets) were also assessed. Among articles related to community retail food environment (*n* = 72), some specifically assessed either ‘unhealthy’ (28%, *n* = 20; explicitly identified as such by the researchers) [63, 64, 94, 98, 102, 103, 110, 116, 121, 124, 130, 132, 134–136, 144, 151–153, 156], ‘healthy’ (17%, *n* = 12; explicitly identified as such by the researchers) [53, 54, 104, 106, 109, 120, 125–128, 142, 143] food outlets or both (7%, *n* = 5) [118, 119, 133, 146, 148]. Thirty-five articles (49%) included various types of food outlets that were not explicitly categorize as ‘healthy’ or ‘unhealthy’ [40, 41, 50, 55, 58, 65, 66, 68, 95–97, 99, 100, 105, 108, 111–113, 115, 117, 122, 123, 129, 131, 137–141, 145, 147, 149, 150, 154, 155]. A ratio of ‘healthy’ or ‘unhealthy’ outlets to total food outlets was calculated in 18% of articles (*n* = 13) [53–55, 104, 109, 118–121, 142, 148, 152, 153]. For studies assessing the consumer retail food environment, food availability, food prominence and food variety were the most assessed outcomes (Table 3).

### Characteristics of articles assessing the food marketing domain

#### Jurisdiction and settings

The Food Marketing environment was most frequently assessed at the national level (53%) (Table 4). A total of 13 different settings, media and techniques through which food marketing occurs were evaluated, the 3 most popular being television (35%), digital media (22%), including in-text message, social applications and websites, and food packaging (20%).

#### Methods and outcomes

Across all settings, in-store data collection (25%), commercial databases (20%), questionnaires (16%) and online audits (12%) were the most popular methods to collect data on food marketing (Table 4). In-store data collection was mainly used for documenting marketing on food packages, and in food stores or restaurants; commercial databases were a common data source for television or digital settings; questionnaires (self-administered, mostly web-based) were used across all settings; and online audit was a method used mainly for digital settings, and to a lesser extent, for assessing marketing on packaged food items on grocery store websites. For recreation sports settings, movie theatres and schools, observational audits were frequently conducted. The research on food marketing included in this review heavily focused on children (69%, *n* = 35) [74–77, 84, 85, 88–90, 157–167, 176–179, 183–193], while adolescents (24%, *n* = 12) [83, 84, 165, 166, 168–171, 187, 190–192] and preschoolers (14%, *n* = 7) were less represented [163, 165, 166, 185, 186, 188, 189]. The age range defining those groups could vary across publications or not be explicitly stated. Other Marketing articles focused on adults or parents (20%, *n* = 10) [165, 166, 169, 171–174, 179, 182, 193], and in 12% (*n* = 6) of publications, no specific group was identified [86, 87, 91, 92, 175, 180].

while adolescents (24%,“Exposure” to food marketing was documented in 78% of publications (Table 4). Of these, 78% (*n* = 31) assessed “potential exposure”[74, 76, 83, 85–87, 91, 92, 160–163, 165–167, 170, 174–181, 184–187, 191–193], representing advertisements that may have been seen by an individual in a specific media/setting [265], 12% (*n* = 6) assessed “actual exposure”[169, 171–173, 182, 190], which captures advertisements that have actually been viewed by an individual [265], and 8% (*n* = 3) assessed both [168, 188, 189]. The types of foods marketed or that would be permitted to be marketed to children according to nutritional criteria (53%), the ‘healthiness’ of foods marketed or that would be permitted to be marketed to children (41%), mainly assessed using nutrient profiling models, and the power of marketing or marketing techniques (39%), referring to the content, design and execution of the marketing message, were also popular outcomes [266, 267]. To a lesser extent, the nutrient content of food marketed (25%) and food and beverage companies that advertised (22%) were also assessed.

### Characteristics of articles assessing the food composition domain

#### Jurisdiction and settings

For most publications, Composition was primarily assessed at the national level (88%) (Table 5). Food supply monitoring predominantly involved foods and beverages available at grocery retailers (86%) and to a lesser extent, at restaurants (14%).

#### Methods and outcomes

Composition data mainly came from research databases (93%). To develop these databases, nutrition information of products was collected from products in stores (79%, *n* = 31) [71, 73, 74, 76–82, 88, 89, 93, 194–209, 218, 219], online (18%, *n* = 7) [75, 210–215] or both (3%, *n* = 1) [216].

As shown in Table 5, the nutrient content of foods (88%) was the most common outcome assessed, followed by the ‘healthiness’ of foods (31%) using various nutrient profiling models [268], including the Food Standards Australia New Zealand – Nutrient Profiling Scoring Criterion [269] and the Health Star Rating system [270], with fewer studies using criteria developed by Health Canada, such as the labelling thresholds for sodium, saturated fat and total sugars that were proposed as part of previously pending federal front-of-package nutrition labelling regulations in Canada [271]. Among publications that monitored the nutrient content of foods and beverages (*n* = 37), 70% (*n* = 26) examined nutrients per 100 g (or mL) [72, 73, 75, 78, 79, 88, 89, 194–196, 198–201, 203, 205, 208–210, 212–214, 216–219], 49% (*n* = 18) per serving or portion [70, 75, 80–82, 89, 194, 200, 202–204, 208, 212–216, 219], and more than 1 unit of analysis were used in 32% (*n* = 12) of publications [75, 89, 194, 195, 200, 203, 208, 212–214, 216, 219]. Other units of analyses included nutrient content per kilocalorie, per 100 kilocalories, per food item or per 50 g, and nutrient content per serving using national reference other than the Canadian one. Overall, 6 publications considered sales data to select food products to include in the study (e.g., products representing more than 80% of market share within a food category) [70, 204, 208, 210, 216] or to assess the comprehensiveness of the set of food products included in a database (for comparison/validation purposes) [217].

### Characteristics of articles assessing the food provision domain

#### Jurisdiction and settings

Most articles in the Provision domain assessed food provision at the provincial/territorial level (71%) (Table 6). School settings were most frequently evaluated (67%), followed by recreation and sport settings (24%), whereas childcare (7%) and healthcare settings (2%) were seldom represented.

#### Methods and outcomes

The 3 primary methods to collect data were observational audits (60%), self-reported questionnaires (38%) and interviews with key stakeholders (26%) (Table 6). Articles often used more than 1 type of data collection methods and various instruments were used to assess food environments in school settings, for example the COMPASS School Environment Application [65, 66, 68, 221], the School Health Policies and Program Survey [222–224], and the Health Behaviour and School-aged Children survey [64, 220]. In other articles, data collection tools were developed by research teams (some of those articles related to the same study) [63, 223, 225–229, 247–249].

Among outcomes frequently reported were food availability (62%), healthy eating initiatives or practices in schools (43%; e.g., cooking classes for student, nutrition committee), adherence to provincial mandatory nutrition policies (e.g., proportion of schools or vending machines meeting the policy standards) or implementation of voluntary provincial guidelines (38%; e.g., actions or initiatives that were implemented in response to the guidelines) and the ‘healthiness’ of foods provided in public settings (36%) (Table 6).

### Characteristics of articles assessing the food prices domain

#### Jurisdiction and settings

Assessment of food prices occurred at various jurisdictional levels, but most frequently at the regional (33%), provincial/territorial (27%) and municipal levels (27%) (Table 7). Food products that were monitored for prices were most frequently items offered in grocery stores (80%) with fewer evaluations of the cost of meals or foods available in convenience stores (20%) and in restaurants (10%).

#### Methods and outcomes

In-store data collection (83%) was the most frequent method to gather information on food prices, including 4 instances where participatory food costing methods were used [52, 71, 253, 254], followed by online data collection (10%) and commercial databases (10%). To support in-store data collection, an instrument commonly used was the Nutrition Environment Measures Survey (NEMS), either the original or adapted versions and for several settings, including for grocery stores (NEMS-S) [272], restaurants (NEMS-R) [273] and convenience stores (NEMS-CS) [274].

In most publications, the price of specific food items or food categories (77%) were assessed, whereas the cost of a diet (13%) and diet affordability (7%), in which household income is accounted for, were less frequently evaluated (Table 7). Among articles assessing diet cost (*n* = 4), either the cost of a healthy diet (75%, *n* = 3) [53, 71, 255], based on nutrient-based and food-based dietary guidelines of a country, or the cost of a current diet (25%, *n* = 1) [252], reflecting food commonly consumed, was assessed [275]. Food pairs comparison refers to the comparison of the cost of pairs of similar items with a difference in nutrient content (e.g., whole wheat vs white pasta) or group of ‘healthier’ items and their ‘less healthy’ counterparts [275]. This outcome was included in 30% of articles, namely those that used the NEMS-S. Among articles that reported on prices of specific foods or food groups (*n* = 18), the price per unit of weight (56%; including price per kilogram, price per 100 g and price per pound), the price per unit or piece (33%) and the price per serving (28%) were the most frequent unit used. In 6 publications, more than 1 unit of analysis were employed [49, 50, 56, 70, 71, 256].

### Characteristics of articles assessing the food labelling domain

#### Jurisdiction and settings

All publications refer to the assessment of food labelling at the national level, and grocery stores were the only setting identified (Table 8).

#### Methods and outcomes

In-store data collection was the sole method used for publications related to Labelling (Table 8). Various systems were used to classify food labels or claims on packaging, the most popular being criteria found in Canadian regulations such as in the Canadian Food and Drug Regulations on nutrition labelling, nutrient content claims and health claims and the Canadian Food Inspection Agency’s Guide to Food Labelling and Advertising [276–281]. For most Labelling articles (53%), a label classification system was not applicable or not mentioned.

A large number of articles examined the types of claims or symbols present on food packages (93%) based on existing criteria or based on other characteristics (e.g., regulated/unregulated claims), and the types of food products carrying claims or symbols (80%). The ‘healthiness’ of foods carrying claims was an outcome in 40% of articles, either those assessing the eligibility of certain foods to carry or not carry a claim based on defined criteria, or those directly assessing the ‘healthiness’ of foods using a nutrient profiling system (e.g., Food Standards Australia New Zealand – Nutrient Profiling Scoring Criterion).

### Characteristics of articles assessing the food trade and investment domain

The 2 articles related to Food Trade and Investment assessed the impact of the Canada–United States Free Trade Agreement (CUSFTA), which entered into force in 1989 and the North American Free Trade Agreement (NAFTA), that entered into force in 1994, and superseded CUSFTA [282, 283]. Both studies used a natural policy experiment approach and applied synthetic control methods, which included creating a control group using a weighted combination of comparison countries that are similar to Canada but that are not exposed to the trade agreement analyzed with which to compare Canada’s outcomes [282]. International (i.e., Food and Agriculture Organization Corporate Statistical Database (FAOSTAT), World Bank World Development Indicators) and American (i.e., United States (US) Department of Agriculture, US Bureau of Economic Analysis) data sources were used. The evaluation of CUSFTA investigated the impact on calorie availability in Canada from 1978 to 2006 via increases in U.S. food exports and investment in Canada’s food and beverage sector. The evaluation of NAFTA, and the second one examined the effect of tariff reductions for food and beverage syrups containing high-fructose corn syrup (HFCS) on the presence of HFCS in the food supply.

### Equity considerations in the assessment of the food environment

**Fig. 3:**
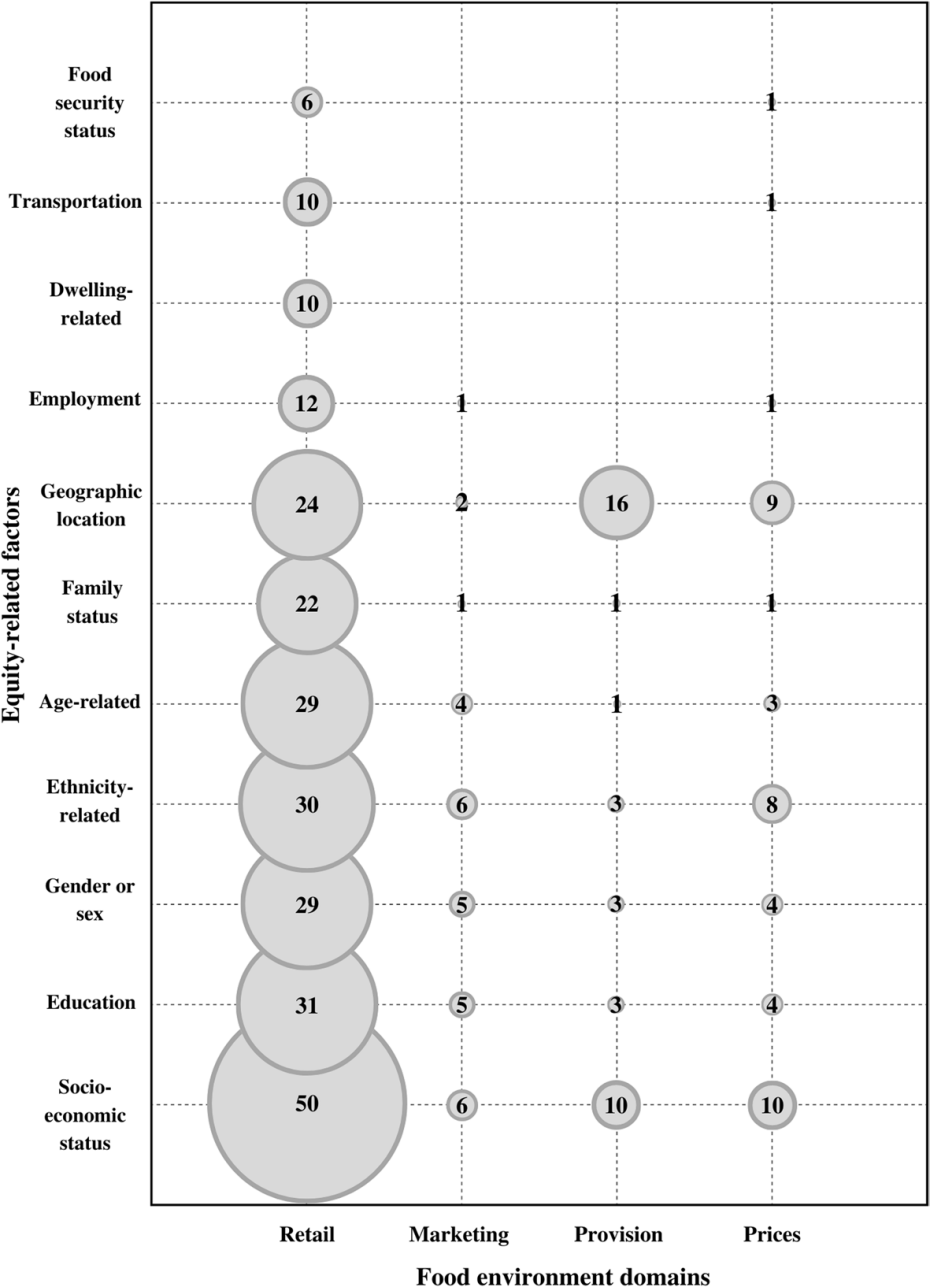
Number of articles accounting for equity in the assessment of food environments, by factor and domain in which equity aspects were considered. Notes: Socioeconomic status included factors related to income such as annual household income, the percentage of residents in a household living below the low-income cut-off, and socioeconomic status more generally (when no definition was provided). Examples of ethnicity-related factors included cultural or racial group, period of arrival in Canada, immigration and aboriginal status. Family status included factors related to parenthood status, number of children, marital status and household size. Geographic location included factors related to rural or urban location, population density or centres and remoteness. Examples of dwelling-related factors included homes needing major/minor repair, average dwelling value, home ownership, and residential instability. Transportation included factors related to car ownership and use of transit

Overall, 108 studies (49%) accounted for equity-related factors in at least 1 domain. Equity considerations were observed in articles related to the Retail (91%, *n* = 81), Prices (70%, *n* = 21), Provision (43%, *n* = 18) and Marketing (22%, *n* = 11) domains. None of the articles studying Labelling, Composition or Trade accounted for equity. The bubble chart in Fig. 3 illustrates, among the 4 domains in which equity aspects were considered, the number of articles, represented by the size of the bubbles, accounting for equity in the assessment of food environments, by equity-related factors and domains. Factors related to socioeconomic status, education, gender or sex, ethnicity, age, family status and geographic location were captured in at least one article. Employment status was included in publications related to Retail, Marketing and Prices. Factors associated with transportation (e.g., car ownership, use of transit) and food insecurity status were included in publications related to Retail and Prices, and only articles related to Retail accounted for aspects associated with residential dwelling (e.g., homes needing major/minor repairs, average dwelling value, home ownership).

Among all articles included in this review, 3% (*n* = 7) [67, 71, 72, 108, 182, 255, 257] were conducted in remote and/or northern communities. Of these articles, 2 assessed Prices and Composition in a First Nation community in Ontario [72] or in 6 Inuit communities of western Canadian Arctic [71], and 1 article assessed Retail and Provision (healthcare setting) in a reserve community in northern Saskatchewan [67].

Overall, 28 publications accounted for equity-related factors using indices that combined multiple socioeconomic dimensions [49, 55, 60, 63, 98, 103, 104, 113, 115, 117, 118, 120–123, 131, 132, 134, 142, 144, 147, 150, 153, 192], and most were publications related to the Retail domain (*n* = 23) [49, 55, 60, 63, 98, 103, 104, 113, 115, 117, 118, 120–123, 131, 132, 134, 142, 144, 147, 150, 153]. Commonly used indices were material and/or social deprivation indices such as the Pampalon index [284]. In some instances, an index was derived or constructed for a specific area, such as the Ontario Marginalization Index derived from the Canadian Marginalization Index [120, 121] or the Vancouver Area Neighbourhood Deprivation Index, constructed from variables obtained from the Canadian census [131, 192].

## Discussion

This analysis of 220 articles published between 2010 and 2021 showed that the 7 food environment domains included in the INFORMAS monitoring framework have been examined in Canada. The number of studies identified suggests that research into food environments has been growing in recent years in Canada, a trend observed through other reviews [23, 24]. Among articles included in our review, 1 in 5 incorporated multiple domains or angles into their results. This underscores the interconnectedness and overlap of the food environment domains as conceptualized in the INFORMAS framework. For example, the Retail food environment is at the confluence of other domains as it could encompass the assessment of food price, food promotion, food placement and food availability [38], thus touching upon elements of Prices, Marketing and Composition. Similarly, a study in a school, classified in the Provision domain as per the INFORMAS framework, could touch upon elements of retail, pricing, composition and marketing. As highlighted in the method section, this aspect of overlapping has to be considered when interpreting the resulting. However, this also points to the opportunity for “joined-up” approaches for interdisciplinary work to advance our comprehensive understanding of food environments at a system-level, and an opportunity for cross-learning and methods development across policy areas. As research methods continue to develop and evolve, evaluating settings as micro-environments incorporating multiple food environment elements will contribute to our holistic understanding of how these environments are evolving.

### Understudied food environments domains

Food Trade and Investment, Food Labelling and, to a lesser extent, Food Prices are domains that are understudied in Canada. Although the literature related to Trade and Investment from a food environment perspective is growing, comprehensive monitoring of the impacts of international trade and investment agreements on food environments is limited [285, 286]. This is an important gap, given that trade agreements can have profound implications for global and national food systems and significantly influence the availability, quality and affordability of foods available in food retail environments, which in turn can influence population-level dietary patterns and health [287].

Similarly, the few studies examining Labelling represent an important gap, given that the Canadian government recently announced new front-of-pack labelling regulations, requiring a symbol on the front panel of packaged food and beverage products if they are high in nutrients of concern (i.e., saturated fat, sugars and sodium), required to be fully implemented by industry by 2026 [288]. Monitoring labelling on product packages in the Canadian food supply is a priority to evaluate the impact of this policy, and how this could change the nutrition information environment in Canada.

Food prices and cost of diet remain critical areas of inquiry. Food insecurity is a serious and persistent public health issue in Canada. In 2021, 15.9% of households representing 5.8 million Canadians, excluding those living in territories or on Indigenous reserves, reported having experienced some level of food insecurity in the previous year [289], and this level has increased compared to the level reported in 2017–2018 [290]. As rising food costs due to inflation exacerbates food insecurity and impact population groups differently (e.g., households with low income or Indigenous Peoples) [289, 291], tracking food prices and the costs of healthier diets on a regular basis can help governments implement policies and actions to facilitate access to healthier foods for all. There is also a need for researchers to develop price monitoring tools or methods that are agile, easily implementable and adaptable to dynamic or unpredictable circumstances.

### Underrepresented settings in the Canadian food environment research

The compiled articles related to Composition and Prices focused mainly on foods and beverages offered in grocery stores, with far fewer examining the costs of foods in restaurant settings. In 2019, Canadians have spent a significant amount of their household food budget on restaurant foods prepared outside the home [292], and national data suggest that more than half (54%) of Canadians eat out at least once per week [293]. This amount has been increasing since 2019 and the pandemic of coronavirus disease 2019 (COVID-19) in 2020 is likely to have accelerated this trend. Considering that greater consumption of foods prepared outside the home has been associated with poorer diet quality, including increased energy intakes and consumption of nutrients of concern [294, 295], monitoring the quality of foods in Canadian food service outlets is of increasing importance. Moreover, research evaluating and comparing the prices of ‘healthy’ and ‘less healthy’ options and investigating menu labelling practices of restaurants, in both brick and mortar and online environments, could contribute to evaluating whether food service outlets in Canada foster healthy choices. In addition, some efforts should be invested in capturing nutritional composition of foods offered in independent and non-franchise restaurants, which might represent a greater proportion of food outlets in some more rural regions. Indeed, literature captured in this review that reported on Composition in restaurants settings included primarily large restaurant chains.

There was also a paucity of evaluation of food environments in healthcare settings, with only 1 publication examining this setting among First Nations women on a reserve community in northern Saskatchewan [67]. Despite the growing acknowledgment that health institutions have a responsibility to lead by example by ensuring that the food served or sold to outpatients, staff, and visitors contributes to healthy diets, their food environments may be suboptimal [296]. The paucity of research that assessed the food environments in healthcare settings in Canada makes it unclear how those institutions are doing in this regard and what are the specific areas that need to be targeted for improvements. In addition, although school food environments were frequently assessed, little is known about the quality of foods offered to infants and toddlers in daycare institutions in Canada. Young children may spend between 35 and 45 h a week in childcare centres, and the meals and snacks they consume may contribute significantly to their daily recommended intakes of foods and nutrients [297, 298]. Therefore, monitoring of the childcare food environment is warranted to ensure these settings contribute to the development and encouragement of healthy eating patterns early in the life course.

Beyond digital marketing, few publications examined digital food environments. Among articles included in this review that assessed Labelling, all were conducted in stores, and none examined food labelling components in online settings, such as e-grocery retail environment or on online food delivery service platforms. In recent years, digital technologies have been integrated to the various components of the food systems, including marketing, distribution and consumption [299, 300]. Digitalization of the food environments is occurring at a fast pace and is becoming a central issue in public health. Indeed, food delivery applications, meal kit services, and online ordering of grocery foods are used by a significant proportion of Canadians. Research has shown that 29%, 20% and 16% of Canadians were using those services respectively, as of 2019 [301], and the COVID-19 pandemic is likely to have increased these proportions. Hence, monitoring those environments to better understand their influence on food-related behaviours and eating practices is of tremendous importance. Similarly, research examining product information in online retail environments, including e-grocers and online food ordering services, remains limited and is an area for further inquiry in order to effectively regulate these environments. As retail environments continue to proliferate in a global market, this remains an area of priority.

### Heterogeneity of food environment research methods

Some commonalities were observed in data collection methods across domains. For example, observational audits were used to evaluate food environments for the Retail, Provision, Prices, Labelling and Marketing (specifically for packaging, stores, restaurants, schools, recreation centres and movie theaters), and questionnaires were commonly used to assess foods provided in publicly funded settings and food marketing on television or digital platforms. However, a vast selection of measures has been employed within domains and even settings. For example, the results from articles examining school food environments demonstrate that an extensive variety of instruments were used to assess school food environments, resulting in similar concepts being measured and reported in different ways. Food prices were reported using multiple price metrics (e.g., price per kilogram, price per pound, price per serving, price per 100 kcal), and 15 different buffers sizes were applied to measure the distribution of food outlets when measuring the consumer retail food environment, which limits comparability across studies. In Retail articles, there was heterogeneity in the classification of food store types (e.g., some used systems such as the Standardized International Classification codes, and in many instances, no specific classification system was reported) as well as in the definition of ‘healthy’ or ‘unhealthy’ food stores. For example, grocery stores were considered as sources of ‘unhealthy’ foods in some publications or as ‘healthy’ food outlets in others. The use of a combination of methods and approaches to study and evaluate food environments has also been observed in previous research [22–24, 302].

Heterogeneity in methods and measures would be expected in this review, as it compiled a wide variety of food environment research that encompassed various components and settings. This wide array of measures can be explained by the multiple definitions and interpretations of food environments that exist [25], the variety of research objectives, and the interdisciplinary nature of this research field (e.g., food and nutrition, public health, geography and urban research). Variability is also, to a certain extent, necessary to ensure that measurements are specific to and appropriate for the food environment context that is evaluated. Moreover, ongoing technological advancements continually allow for advances in existing methods (e.g., automated data collection, or using data collection apps to reduce human errors related to data inputting from paper-based inventory tools), which likely added to the heterogeneity of methods and measures observed in this review. However, variability can make the comparison of research results across studies difficult and may lead to inconsistent research outcomes and results when investigating relationships between food environments and diet- or health-related outcomes.

The current review underscores the potential to harmonize elements of methods and metrics, both within and across domains or settings, to support comparison over time and across jurisdictions [4]. While technological advancements will likely continue to provide more efficient ways to collect and analyze data, better harmonization of *what* is measured and *how* it is reported will allow for stronger inferences about the quality of food environments to better inform policy action and evaluation. For example, this may include more standardized sampling methods and definition of concepts such as buffers, more consistent application and description of criteria for determining the healthfulness of foods or settings, improved fieldwork practices for audits across settings or domains, and greater consistency in reporting outcomes across studies, among others. Previous work has similarly called for standardized metrics and indicators [21], and for flexibility to adapt standardized approaches to local context [4, 303]. The INFORMAS research protocols [4], which were developed for the purposes of standardized, rigorous food environment measurements across countries, are an example of how a core set of harmonized methods can be adapted to various contexts over time and can contribute to the selection and development of tools that will support the collection of high-quality data across geographies and over time. Future work could consider examining how such approaches to harmonization have been used in food environment research.

### Equity lens applied to Canadian food environment research

Equity-related factors were only examined in half of articles, mostly in the Retail environment. While some areas of inquiry are less amenable to examining equity-related factors, such as Labelling and Composition, many studies may have missed valuable opportunities to examine how food environment exposures may differ between population segments (e.g., socioeconomically disadvantaged groups, isolated communities). Previous research also identified the need to pursue food environment research in specific communities, such as indigenous or rural communities [20]. A greater attention to health equity considerations would also be of major importance for domains such as food trade [304, 305]. Indeed, in research examining the influence of trade factors on food environments, applying an equity lens could help understand how the dynamics of international trade and investment agreements shape global inequities in access to healthy foods, particularly between high- and low-income countries. As policies to improve the quality of food environments continue to be developed and implemented in Canada, understanding how these policies may differentially impact various groups, and how they may reduce or exacerbate existing inequities is paramount [306, 307].

An important and related finding is the low representation of studies conducted in remote or northern regions and the assessment of unconventional food environments found in those regions. Of the 7 articles reporting on research in northern or remote regions [67, 71, 72, 108, 182, 255, 257], only 2 included an assessment of foods procured from the land through harvesting, fishing and hunting in Indigenous communities [72, 257]. Indigenous food environments are a unique area of research, as Indigenous populations often rely on both traditional and market foods [72]. Non-traditional market food items are often more expensive, energy dense and highly processed, contributing to food insecurity, poor nutrition and high rates of dietary-related diseases observed among Indigenous populations [308–310]. The paucity of data on Indigenous food environments highlights the potential for research to be undertaken with these communities, developing Indigenous-informed methods to examine these unique and unconventional food environments.

### Implications for future food environment research in Canada and globally

Table 9 summarizes the implications of the findings of this review for future food environments research in Canada. This review has enabled identification of gaps in policy areas that have been identified as necessary and relevant to support healthier food environments, and therefore represent key areas of inquiry to guide future research to improve food environments in Canada. Importantly, this review also identifies a number of active research areas (Food Retail, Food Marketing, Food Composition, Food Provision) which are no less important in informing policy development and evaluation, and ongoing research is warranted. These priorities and recommendations are not ranked.

**Table 9:** Implications for future food environment research in Canada

• As food environments are impacted by multiple policy domains that synergistically shape population dietary patterns, a holistic monitoring of Canadian food environments, with greater efforts to monitor Food Trade and Investment, Food Labelling and Food Prices, should be a priority
• Greater efforts should be invested in the assessment of food environments in underrepresented settings such as the food service sector, healthcare and childcare settings as well as in the rapidly developing digital food environment
• Greater representation of alternative food systems (e.g., food systems of Indigenous Peoples) and of the various levels of the food system (e.g., independent and non-franchise restaurants or food retailers, food environments in rural communities) should be an aim of future research
• Equity considerations should be integrated within all food environment research domains to understand how food environment exposures may differ between population sub-groups, including Indigenous communities
• While research approaches will always be tailored to specific research objectives there may be common elements of food environment research methodologies and approaches across food environment domains that can be better harmonized to support comparisons over time and across jurisdictions
• There is a need for ongoing monitoring of the Canadian food environments across all policy domains as the provincial and federal governments implement policy actions to support healthier food environments (e.g., Health Canada’s Healthy Eating Strategy; [6] the proposed *Child Health Protection Act*, Bill C-252; the Newfoundland and Labrador's tax on sugary drinks [30])

A major gap in the literature identified is a lack of studies examining digital food environments. In the rapidly changed food environment landscape where online purchasing and exposure to marketing are likely to only increase, tools and methodologies that rigorously monitor and evaluate digital food environments will play a key role in informing policy development and evaluation.

### Strengths and limitations

There are several strengths to our study. First, this review is, to our knowledge, the first to compile the Canadian literature on the evaluation of food environments in Canada, and to examine both English and French literature. The broad definition of food environments, coupled with the use of an internationally-implemented framework with multiple food environment domains to develop the search strategy is also an important strength. Consideration for and analysis of equity-related factors accounted for in Canadian food environment research is another strength. However, several limitations should be recognized. First, the study only examines the Canadian literature, which may not represent the research being conducted in other countries and contexts. Additional examinations of food environment research methods and comparisons over countries in the types and usages of methods could help further inform methods development. However, by focusing our review within a single national jurisdiction with a large literature on food environments, we think this work provides insight on practical knowledge creation and has important knowledge use implications (e.g., to identify the most frequently used measures, and where we should go from here in expanding this field of research, areas for local researchers collaboration and research integration, overlaps between food environment domains and food environment decision-making at the national and subnational level). Other limitations are largely related to the use of a rapid review approach which is more limited in scope than other review types. The documents included in the review were limited to peer-reviewed articles and excluded any potentially relevant grey literature. For example, food costing often occurs at the provincial or local levels and the results of those assessments are reflected in grey literature rather than in the peer-reviewed literature. Because the objective of the study was not to examine the robustness of the evidence but rather to provide an overview of the literature on food environment measures and identify gaps in the literature, the quality of included studies was not assessed, as is common practice for scoping or rapid reviews [20, 33, 34]. For similar reasons, no formal analysis of publications that tested psychometric properties of tools used was performed. Furthermore, examining the relationship between the food environment and individuals’ dietary patterns or health outcomes was outside the scope of the review. Data extraction and coding were mostly completed by 1 reviewer, allowing room for subjectivity, but frequent discussions with the lead author helped clarify coding criteria and ensure thoroughness. Finally, the review was based on number of papers published, and not number of studies conducted. Some articles reported on data from the same study using multiple approaches. This process was deemed appropriate as the aim of this rapid review was to compile the different metrics and methods used to assess food environments and not report on the outcomes themselves; however, it may falsely create the appearance of more research being conducted in some policy domains.

## Conclusion

While food environment research in Canada has grown over the past decade, a number of gaps remain that prevent a holistic and systems-level analysis of the quality of food environments in Canada. As food environments are impacted by multiple policy domains that synergistically shape population dietary patterns, filling these research gaps using rigorous research methods will provide a more comprehensive understanding of the quality of food environments in Canada and opportunities for policy action. This review may help inform researchers of the methods that have been recently implemented to assess the various elements of food environments. The wide range of fields and disciplines conducting research on food environments reflects the need for collaboration and interdisciplinary work to further this field of inquiry. As Canada continues to implement policies to improve the quality of food environments in order to improve dietary patterns, targeted research to address identified gaps and harmonize methods across studies will help evaluate policy impact over time.

### Supplementary Information

**Additional file 1. **PRISMA Checklist. This file contains the Preferred Reporting Items for Systematic Reviews and Meta-Analyses (PRISMA) checklist used to support adequate reporting (for items applicable to the present work).

**Additional file 2. **Full search strategies for all databases Web of Science, CAB Abstracts and Ovid MEDLINE databases. This file contains the search strategies used for this review for each of the databases.

**Additional file 3. **Screening tool. This file contains the screening tool used in this review to guide the screening process and ensure consistency.

## Data Availability

The datasets used and/or analysed during the current study are available from the corresponding author on reasonable request.

## Abbreviations

COVID-19: Coronavirus disease 2019
CUSFTA: Canada–United States Free Trade Agreement
EPOI: Enhanced Points of Interest
FAOSTAT: Food and Agriculture Organization Corporate Statistical Database
FOPL: Front-of-Package Label
GPS: Global Positioning System
HFCS: High-fructose corn syrup
INFORMAS: International Network for Food and Obesity Research, Monitoring and Action Support
IOM: Institute of Medicine
NAFTA: North American Free Trade Agreement
NEMS: Nutrition Environment Measures Survey
NEMS-CS: Nutrition Environment Measures Survey for Convenience Stores
NEMS-R: Nutrition Environment Measures Survey for Restaurants
NEMS-S: Nutrition Environment Measures Survey for Grocery Stores
PRISMA: Preferred Reporting Items for Systematic Reviews and Meta-Analyses
US: United States
WHO: World Health Organization
