# Supplementary material for: Food environment research in Canada: a rapid review of methodologies and measures deployed between 2010 and 2021
Source: Int J Behav Nutr Phys Act. 2024 Feb 19;21:18. doi: 10.1186/s12966-024-01558-x (PMC10875887; doi:10.1186/s12966-024-01558-x)
Supplement: Supplementary file 2 — Additional file 2. Full search strategies for all databases Web of Science, CAB Abstracts and Ovid MEDLINE databases. This file contains the search strategies used for this review for each of the databases. [file 12966_2024_1558_MOESM2_ESM.docx]

**Full search strategies for all databases Web of Science, CAB Abstracts and Ovid MEDLINE databases**

**Search strategy - Web of Science**

Number of results (June 17, 2021): 858

TOPIC: ((assessing or assess or analysis or comparison or examining or scan or checklist* or evaluate or evaluation or evaluating) NEAR/3 ("nutritional value*" or nutrient* or "nutritional quality" or nutrition or "food environment*" or "food advertising" or "in-store environment*" or "nutrition environment*" or foodscape or "food supply" or "food composition" or "food healthfulness" or "food content*" or "food process*" or "food packag*") ) AND TOPIC: ((Canad* or "british columbia" or "colombie britannique" or alberta* or saskatchewan or manitoba* or ontario or quebec or "new brunswick" or "nouveau brunswick" or "nova scotia" or "nouvelle ecosse" or "prince edward island" or newfoundland or labrador or nunavut or nwt or "northwest territories" or yukon or nunavik or inuvialuit) ) OR TOPIC: ((food* or beverage* or nutrition* or snack* or "food environment*") NEAR/3 (outdoor or "digital market*" or market* or promotion or packag* or "pre-packg*" or "front-of-pack or label*" or information or ingredient* or appeal* or ads or brand* or logo or publicity or "social media" or TV or radio or internet or school* or celebrit* or broadcast or sponsorship or "health claim*" or "nutrition* claim*") ) AND TOPIC: ((Canad* or "british columbia" or "colombie britannique" or alberta* or saskatchewan or manitoba* or ontario or quebec or "new brunswick" or "nouveau brunswick" or "nova scotia" or "nouvelle ecosse" or "prince edward island" or newfoundland or labrador or nunavut or nwt or "northwest territories" or yukon or nunavik or inuvialuit) ) OR TOPIC: (("nutrition guideline*" or "nutrition polic*" or "nutrition standard*") near/3 (child* or youth or school* or "publicly funded institution*" or "public sector*" or hospital* or healthcare* or cafeteria or canteen* or store* or preschool* or daycare or "recreation centre" or "sport* centre" or facilitie* or arena or "vending machine") ) AND TOPIC: ((Canad* or "british columbia" or "colombie britannique" or alberta* or saskatchewan or manitoba* or ontario or quebec or "new brunswick" or "nouveau brunswick" or "nova scotia" or "nouvelle ecosse" or "prince edward island" or newfoundland or labrador or nunavut or nwt or "northwest territories" or yukon or nunavik or inuvialuit) ) OR TOPIC: (("diet quality" or "food access*" or "food availability" or availability or affordab* or "food desert" or proximity or "food swamp") near/3 (price* or "healthy food*" or "healthy diet" or cost* or financial* or budget or retail or supermarket* or "grocery store*" or "convenience store*" or "corner store*" or "food store*" or "dollar store*" or "speciality store" or "gas station*" or "fast food*" or restaurant* or bodega or tienda or outlet* or establishment* or premises or neighbourhood or store* or "household expenditure*" or communit*) ) AND TOPIC: ((Canad* or "british columbia" or "colombie britannique" or alberta* or saskatchewan or manitoba* or ontario or quebec or "new brunswick" or "nouveau brunswick" or "nova scotia" or "nouvelle ecosse" or "prince edward island" or newfoundland or labrador or nunavut or nwt or "northwest territories" or yukon or nunavik or inuvialuit) ) OR TOPIC: ((tax* or ban* or staple* or quota* or agreement* or trade or import or importing or export or exporting or tariff* or NAFTA or "north american free trade agreement" or "investment agreement*" or "US Mexico Canada Agreement" or USMCA or "Canada European Union Comprehensive Economic Trade Agreement" or CETA) near/3 (food* or beverage*) ) AND TOPIC: ((Canad* or "british columbia" or "colombie britannique" or alberta* or saskatchewan or manitoba* or ontario or quebec or "new brunswick" or "nouveau brunswick" or "nova scotia" or "nouvelle ecosse" or "prince edward island" or newfoundland or labrador or nunavut or nwt or "northwest territories" or yukon or nunavik or inuvialuit) )

Refined by: LANGUAGES: ( ENGLISH OR FRENCH ) AND COUNTRIES/REGIONS: ( CANADA ) AND [excluding] RESEARCH AREAS: ( FISHERIES OR ENVIRONMENTAL SCIENCES ECOLOGY OR METEOROLOGY ATMOSPHERIC SCIENCES OR INSTRUMENTS INSTRUMENTATION OR ZOOLOGY OR ENERGY FUELS OR MEDICAL INFORMATICS OR SCIENCE TECHNOLOGY OTHER TOPICS OR PHYSIOLOGY OR CRIMINOLOGY PENOLOGY OR WATER RESOURCES OR ENTOMOLOGY OR COMPUTER SCIENCE OR ENGINEERING OR HISTORY PHILOSOPHY OF SCIENCE OR MARINE FRESHWATER BIOLOGY OR OCEANOGRAPHY OR GEOCHEMISTRY GEOPHYSICS OR CARDIOVASCULAR SYSTEM CARDIOLOGY OR FORESTRY OR CELL BIOLOGY OR PLANT SCIENCES OR INFECTIOUS DISEASES OR NUCLEAR SCIENCE TECHNOLOGY OR UROLOGY NEPHROLOGY OR PHYSICS OR GEOGRAPHY OR RESPIRATORY SYSTEM OR AUDIOLOGY SPEECH LANGUAGE PATHOLOGY OR CONSTRUCTION BUILDING TECHNOLOGY OR ONCOLOGY OR BIODIVERSITY CONSERVATION OR REMOTE SENSING OR DERMATOLOGY OR GEOLOGY OR ARCHAEOLOGY OR EVOLUTIONARY BIOLOGY OR INFORMATION SCIENCE LIBRARY SCIENCE OR VETERINARY SCIENCES OR MATERIALS SCIENCE OR TRANSPORTATION OR MECHANICS OR PHYSICAL GEOGRAPHY OR MINING MINERAL PROCESSING OR ORTHOPEDICS )

Indexes=SCI-EXPANDED, SSCI, A&HCI, CPCI-S, CPCI-SSH, ESCI Timespan=2010-2021

**Search strategy - CAB Abstracts**

| **#** | **Request** | **Results (June 15, 2021)** |
| --- | --- | --- |
| 1 | ((assessing or assess or analysis or comparison or examining or scan or checklist* or evaluate or evaluation or evaluating) adj3 (nutritional value* or nutrient* or nutritional quality or nutrition or food environment* or food advertising or in-store environment* or nutrition environment* or foodscape or food supply or food composition or food healthfulness or food content* or food process* or food packag*)).ab,ti. | 12,888 |
| 2 | assessment/ | 74,964 |
| 3 | checklists/ | 7,634 |
| 4 | evaluation/ | 165,682 |
| 5 | comparisons/ | 40,181 |
| 6 | 2 or 3 or 4 or 5 | 277,404 |
| 7 | nutritive value/ | 97,503 |
| 8 | food environment/ | 375 |
| 9 | food advertising/ | 486 |
| 10 | food supply/ | 11,985 |
| 11 | food composition/ | 10,389 |
| 12 | food processing/ | 42,662 |
| 13 | food packaging/ | 9,720 |
| 14 | 7 or 8 or 9 or 10 or 11 or 12 or 13 | 165,825 |
| 15 | 6 and 14 | 6,937 |
| 16 | 1 or 15 | 19,456 |
| 17 | ((food* or beverage* or nutrition* or snack* or food environment*) adj3 (outdoor or digital market* or market* or promotion or packag* or pre-packg* or front-of-pack or label* or information or ingredient* or appeal* or ads or brand* or logo or publicity or social media or TV or radio or internet or school* or celebrit* or broadcast or sponsorship or health claim* or nutrition* claim*)).ab,ti. | 47,536 |
| 18 | exp foods/ | 412,069 |
| 19 | beverages/ or fruit drinks/ or lactic beverages/ or soft drinks/ | 25,536 |
| 20 | snacks/ | 5,316 |
| 21 | food environment/ | 375 |
| 22 | 18 or 19 or 20 or 21 | 414,622 |
| 23 | marketing/ or food marketing/ or social marketing/ or food merchandising/ | 61,952 |
| 24 | health promotion/ or wellness/ | 15,891 |
| 25 | publicity/ or advertising/ or mass media/ | 11,871 |
| 26 | information/ or consumer information/ or nutrition information/ | 20,322 |
| 27 | branding/ | 200 |
| 28 | labelling/ or nutrition labelling/ or quality labelling/ or merchandise information/ | 13,385 |
| 29 | schools/ or elementary schools/ or high schools/ or nursery schools/ or private schools/ or public schools/ | 14,354 |
| 30 | sponsorship/ | 961 |
| 31 | internet/ or telecommunications/ or e-commerce/ | 15,773 |
| 32 | 23 or 24 or 25 or 26 or 27 or 28 or 29 or 30 or 31 | 140,640 |
| 33 | 22 and 32 | 16,149 |
| 34 | 17 or 33 | 58,364 |
| 35 | ((nutrition guideline* or nutrition polic* or nutrition standard*) adj3 (child* or youth or school* or publicly funded institution* or public sector* or hospital* or healthcare* or cafeteria or canteen* or store* or preschool* or daycare or recreation centre or sport* centre or facilitie* or arena or vending machine)).ab,ti. | 223 |
| 36 | nutrition programmes/ or nutrition policy/ | 9,447 |
| 37 | children/ or adolescents/ | 205,781 |
| 38 | youth/ | 12,509 |
| 39 | public sector/ | 8,265 |
| 40 | schools/ or elementary schools/ or high schools/ or nursery schools/ or private schools/ or public schools/ or school food service/ | 14,599 |
| 41 | dining facilities/ or cafes/ or cafeterias/ or community feeding centres/ or public houses/ or restaurants/ | 7,055 |
| 42 | hospitals/ or hospital catering/ | 21,270 |
| 43 | sports centres/ | 445 |
| 44 | vending machines/ | 187 |
| 45 | indoor arenas/ or recreational facilities/ | 2,451 |
| 46 | 37 or 38 or 39 or 40 or 41 or 42 or 43 or 44 or 45 | 253,979 |
| 47 | 36 and 46 | 3,436 |
| 48 | 35 or 47 | 3,577 |
| 49 | ((diet quality or food access* or food availability or availability or affordab* or food desert or proximity or food swamp) adj3 (pric* or healthy food* or healthy diet or cost* or financial* or budget or retail or supermarket* or grocery store* or convenience store* or corner store* or food store* or dollar store* or speciality store or gas station* or fast food* or restaurant* or bodega or tienda or outlet* or establishment* or premises or neighbourhood or store* or household expenditure* or communit*)).ab,ti. | 7,049 |
| 50 | food access/ | 575 |
| 51 | food deserts/ | 121 |
| 52 | 50 or 51 | 627 |
| 53 | prices/ or food prices/ | 47,171 |
| 54 | health foods/ | 1,693 |
| 55 | costs/ or food costs/ | 71,662 |
| 56 | budgets/ or family budgets/ or household budgets/ | 1,590 |
| 57 | retail prices/ | 1,437 |
| 58 | supermarkets/ | 3,569 |
| 59 | fast foods/ | 2,380 |
| 60 | neighbourhoods/ or residential areas/ | 5,471 |
| 61 | food stores/ | 394 |
| 62 | communities/ | 46,985 |
| 63 | 53 or 54 or 55 or 56 or 57 or 58 or 59 or 60 or 61 or 62 | 174,550 |
| 64 | 52 and 63 | 151 |
| 65 | 49 or 64 | 7,160 |
| 66 | ((tax* or ban* or staple* or quota* or agreement* or trade or import or importing or export or exporting or tariff* or NAFTA or north american free trade agreement or investment agreement* or US Mexico Canada Agreement or USMCA or Canada European Union Comprehensive Economic Trade Agreement or CETA) adj3 (food* or beverage*)).ab,ti. | 14,776 |
| 67 | taxes/ | 7,117 |
| 68 | staple/ | 1,001 |
| 69 | import quotas/ or import controls/ or imports/ | 15,772 |
| 70 | exports/ | 24,249 |
| 71 | tariffs/ | 2,060 |
| 72 | trade agreements/ | 2,842 |
| 73 | 67 or 68 or 69 or 70 or 71 or 72 | 44,243 |
| 74 | exp foods/ | 412,069 |
| 75 | beverages/ or fruit drinks/ or lactic beverages/ or soft drinks/ | 25,536 |
| 76 | 74 or 75 | 412,069 |
| 77 | 73 and 76 | 3,346 |
| 78 | 66 or 77 | 17,682 |
| 79 | 16 or 34 or 48 or 65 or 78 | 100,756 |
| 80 | alcoholic beverage*.ab,ti. | 4,260 |
| 81 | 79 not 80 | 100,209 |
| 82 | (Canad* or british columbia or colombie britannique or alberta* or saskatchewan or manitoba* or ontario or quebec or new brunswick or nouveau brunswick or nova scotia or nouvelle ecosse or prince edward island or newfoundland or labrador or nunavut or nwt or northwest territories or yukon or nunavik or inuvialuit).ab,ti. | 174,799 |
| 83 | 81 and 82 | 2,175 |
| 84 | limit 83 to ((english or french) and yr="2010 - 2021") | 1,043 |

**Search strategy - All Ovid MEDLINE(R)**

| **#** | **Request** | **Results (June 15, 2021)** |
| --- | --- | --- |
| 1 | ((assessing or assess or analysis or comparison or examining or scan or checklist* or evaluate or evaluation or evaluating) adj3 (nutritional value* or nutrient* or nutritional quality or nutrition or food environment* or food advertising or in-store environment* or nutrition environment* or foodscape or food supply or food composition or food healthfulness or food content* or food process* or food packag*)).ab,kw,ti. | 5,597 |
| 2 | ((food* or beverage* or nutrition* or snack* or food environment*) adj3 (outdoor or digital market* or market* or promotion or packag* or pre-packg* or front-of-pack or label* or information or ingredient* or appeal* or ads or brand* or logo or publicity or social media or TV or radio or internet or school* or celebrit* or broadcast or sponsorship or health claim* or nutrition* claim*)).ab,kw,ti. | 25,743 |
| 3 | ((nutrition guideline* or nutrition polic* or nutrition standard*) adj3 (child* or youth or school* or publicly funded institution* or public sector* or hospital* or healthcare* or cafeteria or canteen* or store* or preschool* or daycare or recreation centre or sport* centre or facilitie* or arena or vending machine)).ab,kw,ti. | 305 |
| 4 | ((diet quality or food access* or food availability or availability or affordab* or food desert or proximity or food swamp) adj3 (price* or healthy food* or healthy diet or cost* or financial* or budget or retail or supermarket* or grocery store* or convenience store* or corner store* or food store* or dollar store* or speciality store or gas station* or fast food* or restaurant* or bodega or tienda or outlet* or establishment* or premises or neighbourhood or store* or household expenditure* or communit*)).ab,kw,ti. | 8,854 |
| 5 | exp Food/ | 1,337,393 |
| 6 | beverages/ or artificially sweetened beverages/ or carbonated beverages/ or coffee/ or drinking water/ or energy drinks/ or "fruit and vegetable juices"/ or milk/ or milk substitutes/ or sugar-sweetened beverages/ or tea/ or teas, herbal/ or teas, medicinal/ | 103,590 |
| 7 | 5 or 6 | 1,367,822 |
| 8 | 4 and 7 | 915 |
| 9 | ((tax* or ban* or staple* or quota* or agreement* or trade or import or importing or export or exporting or tariff* or NAFTA or north american free trade agreement or investment agreement* or US Mexico Canada Agreement or USMCA or Canada European Union Comprehensive Economic Trade Agreement or CETA) adj3 (food* or beverage*)).ab,kw,ti. | 4,798 |
| 10 | (Canad* or british columbia or colombie britannique or alberta* or saskatchewan or manitoba* or ontario or quebec or new brunswick or nouveau brunswick or nova scotia or nouvelle ecosse or prince edward island or newfoundland or labrador or nunavut or nwt or northwest territories or yukon or nunavik or inuvialuit).ab,kw,ti. | 174,830 |
| 11 | Checklist/ | 7,043 |
| 12 | Nutritive Value/ | 14,653 |
| 13 | Nutrients/ | 3,564 |
| 14 | food handling/ or food packaging/ | 30,227 |
| 15 | 12 or 13 or 14 | 46,591 |
| 16 | 11 and 15 | 8 |
| 17 | 1 or 16 | 5,605 |
| 18 | exp Food/ | 1,337,393 |
| 19 | beverages/ or artificially sweetened beverages/ or carbonated beverages/ or coffee/ or drinking water/ or energy drinks/ or "fruit and vegetable juices"/ or milk/ or milk substitutes/ or sugar-sweetened beverages/ or tea/ or teas, herbal/ or teas, medicinal/ | 103,590 |
| 20 | Snacks/ | 1,592 |
| 21 | 18 or 19 or 20 | 1,367,822 |
| 22 | marketing/ or advertising/ or social marketing/ | 22,655 |
| 23 | Health Promotion/ | 76,542 |
| 24 | product packaging/ or product labeling/ | 4,144 |
| 25 | Food Labeling/ | 4,040 |
| 26 | mass media/ or radio/ or television/ | 26,002 |
| 27 | internet/ or social media/ | 84,447 |
| 28 | schools/ or schools, nursery/ | 42,884 |
| 29 | 22 or 23 or 24 or 25 or 26 or 27 or 28 | 244,914 |
| 30 | 21 and 29 | 12,355 |
| 31 | 2 or 30 | 35,091 |
| 32 | Nutrition Policy/ | 9,851 |
| 33 | child/ or child, preschool/ | 1,974,957 |
| 34 | Adolescent/ | 2,097,282 |
| 35 | schools/ or schools, nursery/ | 42,884 |
| 36 | Food Service, Hospital/ | 4,804 |
| 37 | Public Sector/ | 6,692 |
| 38 | 33 or 34 or 35 or 36 or 37 | 3,143,652 |
| 39 | 32 and 38 | 3,314 |
| 40 | 3 or 39 | 3,449 |
| 41 | 8 or 9 or 17 or 31 or 40 | 47,242 |
| 42 | 10 and 41 | 1,199 |
| 43 | limit 42 to (yr="2010 - 2021" and (english or french)) | 865 |
| 44 | limit 43 to "humans only (removes records about animals)" | 835 |

ERIC <1965 to May 2021>

| **#** | **Request** | **Results (June 16, 2021)** |
| --- | --- | --- |
| 1 | ((assessing or assess or analysis or comparison or examining or scan or checklist* or evaluate or evaluation or evaluating) adj3 (nutritional value* or nutrient* or nutritional quality or nutrition or food environment* or food advertising or in-store environment* or nutrition environment* or foodscape or food supply or food composition or food healthfulness or food content* or food process* or food packag*)).ti,ab. | 249 |
| 2 | ((food* or beverage* or nutrition* or snack* or food environment*) adj3 (outdoor or digital market* or market* or promotion or packag* or pre-packg* or front-of-pack or label* or information or ingredient* or appeal* or ads or brand* or logo or publicity or social media or TV or radio or internet or school* or celebrit* or broadcast or sponsorship or health claim* or nutrition* claim*)).ti,ab. | 2,370 |
| 3 | ((nutrition guideline* or nutrition polic* or nutrition standard*) adj3 (child* or youth or school* or publicly funded institution* or public sector* or hospital* or healthcare* or cafeteria or canteen* or store* or preschool* or daycare or recreation centre or sport* centre or facilitie* or arena or vending machine)).ti,ab. | 59 |
| 4 | ((diet quality or food access* or food availability or availability or affordab* or food desert or proximity or food swamp) adj3 (price* or healthy food* or healthy diet or cost* or financial* or budget or retail or supermarket* or grocery store* or convenience store* or corner store* or food store* or dollar store* or speciality store or gas station* or fast food* or restaurant* or bodega or tienda or outlet* or establishment* or premises or neighbourhood or store* or household expenditure* or communit*)).ti,ab. | 1,176 |
| 5 | exp food/ | 5,610 |
| 6 | exp food service/ | 2,122 |
| 7 | food standards/ | 715 |
| 8 | nutrition/ or breakfast programs/ or lunch programs/ | 7,980 |
| 9 | exp schools/ | 261,175 |
| 10 | exp hospitals/ | 3,293 |
| 11 | public sector/ | 1,261 |
| 12 | exp dining facilities/ | 699 |
| 13 | exp recreational facilities/ | 2,488 |
| 14 | health promotion/ | 8,456 |
| 15 | 5 or 6 or 7 or 8 | 13,273 |
| 16 | 3 or 9 or 10 or 11 or 12 or 13 or 14 | 275,296 |
| 17 | ((tax* or ban* or staple* or quota* or agreement* or trade or import or importing or export or exporting or tariff* or NAFTA or north american free trade agreement or investment agreement* or US Mexico Canada Agreement or USMCA or Canada European Union Comprehensive Economic Trade Agreement or CETA) adj3 (food* or beverage*)).ti,ab. | 164 |
| 18 | (Canad* or british columbia or colombie britannique or alberta* or saskatchewan or manitoba* or ontario or quebec or new brunswick or nouveau brunswick or nova scotia or nouvelle ecosse or prince edward island or newfoundland or labrador or nunavut or nwt or northwest territories or yukon or nunavik or inuvialuit).ti,ab. | 32,809 |
| 19 | evaluation methods/ | 50,044 |
| 20 | check lists/ | 6,965 |
| 21 | 19 or 20 | 56,256 |
| 22 | nutrition/ or breakfast programs/ or lunch programs/ | 7,980 |
| 23 | 21 and 22 | 180 |
| 24 | 1 or 23 | 419 |
| 25 | food/ | 5,610 |
| 26 | marketing/ or merchandise information/ | 8,461 |
| 27 | health promotion/ or wellness/ | 9,438 |
| 28 | publicity/ or mass media/ | 8,989 |
| 29 | social media/ | 3,782 |
| 30 | High Schools/ or Elementary Schools/ | 51,144 |
| 31 | advertising/ or television commercials/ | 4,240 |
| 32 | 26 or 27 or 28 or 29 or 30 or 31 | 83,268 |
| 33 | 25 and 32 | 899 |
| 34 | 2 or 33 | 2,983 |
| 35 | 5 or 6 or 7 or 8 | 13,273 |
| 36 | 9 or 10 or 11 or 12 or 13 or 14 | 275,268 |
| 37 | 35 and 36 | 3,053 |
| 38 | 3 or 37 | 3,083 |
| 39 | 4 or 17 or 24 or 34 or 38 | 6,459 |
| 40 | 18 and 39 | 137 |
| 41 | limit 40 to ((english or french) and yr="2010 - 2021") | 54 |
