# Supplementary material for: Food environment research in Canada: a rapid review of methodologies and measures deployed between 2010 and 2021
Source: Int J Behav Nutr Phys Act. 2024 Feb 19;21:18. doi: 10.1186/s12966-024-01558-x (PMC10875887; doi:10.1186/s12966-024-01558-x)
Supplement: Supplementary file 3 — Additional file 3. Screening tool. This file contains the screening tool used in this review to guide the screening process and ensure consistency. [file 12966_2024_1558_MOESM3_ESM.docx]

**SCREENING TOOL – RAPID REVIEW**

*Built for citation, title, and abstract screening, and further refine for the full-text screening process.*

1. **Does the citation indicate publication on or after 2010?**
   1. Yes: continue screening
   2. No: stop screening
2. **Does the title or abstract use English or French?**
   1. Yes: continue screening
   2. No: stop screening
3. **Does the title or abstract indicate that the study (or part of it) was conducted in Canada?**
   1. Yes: continue screening
      - If a study is conducted in multiple countries, Canada must be one of those countries for the document to be accepted
   2. No: stop screening
4. **Does the title or abstract indicate that this study is published in a peer-reviewed journal?**
   1. Yes: continue screening
   2. No: stop screening
5. **Does the title or abstract indicate that this is a review, letter, editorial, commentary, correction, proceedings, publication listing abstracts on research work presented at a conference (or meeting abstracts), book, book chapter, news or erratum?**
   1. Yes: stop screening
      - Position statements are excluded unless preceded by a review of the literature
   2. No: continue screening
6. **Does the abstract/text indicate that an evaluation of the food environment and/or ≥1 specific areas of the food environment was conducted?**
   1. Yes (or unsure/unclear during the title and abstract screening process): continue screening
      - Intervention studies are accepted if an evaluation/assessment component (of the food environment or specific areas) is present.
   2. No: stop screening
      - Studies assessing the home food environment are excluded
7. **For studies that evaluate the food composition**: **Does the abstract/text indicate that the evaluation concerns ≥1 of the following settings/aspects: supermarkets, grocery stores, convenience stores, restaurants, packaged foods?**
   1. Yes (or unsure/unclear during the title and abstract screening process): continue screening
      - Food composition must be related to ≥1 of the following nutrients: energy, fat, saturated fat, sodium, sugar, added sugars, fibers or to a nutrient profiling system (multiple nutrients considered)
      - Studies assessing the food composition of packaged foods from nutritional database (e.g., FLIP) are included
      - Studies assessing healthiness of food offered on online delivery platforms are included (it’s essentially restaurants food supply)
      - Studies assessing the nutritional quality of foods in restaurant settings are included
   2. No: stop screening
      - Studies related to food safety or food contamination are excluded
      - Studies related exclusively to alcoholic beverages are excluded
      - Studies related to the composition/content of baby foods only (e.g., perchlorate content, lead, etc.) are excluded
      - Studies related to the content in probiotic strains are excluded
      - Studies looking at composition/content of foods from a sample (not from the actual food environment) are excluded (e.g., a study assessing the protein and carbs content of samples of lentils with different characteristics)
8. **For studies that evaluate the food labelling: Does the abstract/text indicate that the evaluation relates to nutrition or health claims on food packages?**
   1. Yes (or unsure/unclear during the title and abstract screening process): continue screening
      - Studies related to genetically modified food labels are included
      - Studies must assess the **actual** food environment (or specific aspects of it) **already in place**, i.e., the evaluation must be conducted in **real-world setting** (prediction/simulation models, or experimental design, such as mock packages) are excluded)
   2. No: stop screening
      - Studies assessing only the perception/attitude of community actors towards food environment and consumers' behaviors (food intake, food consumption) are excluded
      - Studies related exclusively to alcoholic beverages are excluded
9. **For studies that evaluate food marketing**: **Does the abstract/text indicate that the evaluation concerns ≥1 of the following settings/channels: schools, outdoor, retail, recreation setting, digital, TV, packages, sport sponsorship, other?**
   1. Yes (or unsure/unclear during the title and abstract screening process): continue screening
      - Studies assessing self-reported exposure to marketing are included
      - Studies testing a new tool to assess marketing (in a relevant setting) are included
   2. No: stop screening
      - Studies related exclusively to alcoholic beverages are excluded
      - Studies related to marketing of vitamins, minerals supplements are excluded (because those products are not considered foods)
      - Studies related to health and safety claims of cannabis edibles are excluded
10. **For studies that evaluate the food provision**: **Does the abstract/text indicate that the evaluation concerns ≥1 of the following governmental funded settings: schools (any level), recreation sport settings, hospitals, daycare?**
    1. Yes (or unsure/unclear during the title and abstract screening process): continue screening
       - Studies in post-secondary (university) settings are included
       - Studies related to nutrition policy in schools (e.g., presence or absence AND characterization in terms of nutrition standards for example) are included
       - Studies with self-reported (by principals, teachers, etc.) measures are included only if items measured are **objective and factual in nature** (e.g., availability of fruits and vegetables at schools, ratio of foods in vending machine meeting provincial school nutrition guidelines, presence of written nutrition policy)
       - Studies related to food supplementation, breakfast program or “closed campus policies” are included only if the quality of foods is evaluated (if nutrition standards are applied)
    2. No: stop screening
       - Studies assessing only the perception/attitude of community actors towards food environment and consumers' behaviors (food intake, food consumption) are excluded
       - Studies relating to the evaluation of the food provided to in-patients menus are excluded
       - Studies in school settings assessing the content of lunch boxes brought from home are excluded, unless it is compared with foods offer at school
       - Studies with self-reported (by principals, teachers, etc.) measures are excluded if items measured are subjective in nature (e.g., perceptions regarding the school’s healthy eating initiatives)
11. **For studies that evaluate food retail: Does the abstract/text indicate that the evaluation of the *community* (e.g., location and accessibility of food outlets) or *consumer* (e.g., placement, shelve space of foods items) nutrition environment?**
    1. Yes (or unsure/unclear during the title and abstract screening process): continue screening
       - Studies related to menu labelling in restaurants are included
       - Studies assessing the density of food outlets per X people in general (no setting specified), are included
       - Studies conducted in and around schools, including post-secondary (university) settings, are included
    2. No: stop screening
       - Studies assessing only the perception/attitude of community actors towards food environment and consumers' behaviors (food intake, food consumption) are excluded
       - Studies related exclusively to alcoholic beverages are excluded
12. **For studies that evaluate food prices (relative or absolute, affordability): Does the abstract/text indicate that the evaluation relates to the price of diets, meals and/or foods?**
    1. Yes (or unsure/unclear during the title and abstract screening process): continue screening
    2. No: stop screening
       - Studies related exclusively to alcoholic beverages are excluded
       - Studies related to the prices of cannabis or cannabis food products are excluded
13. **For studies that evaluate food trade and investment**: **Does the abstract/text indicate that**

- **Both trade indicators and food environment indicators are monitored/evaluated?**

**AND**

- **The study is about the impacts on Canadian domestic food environment?**
  1. Yes (or unsure/unclear during the title and abstract screening process): continue screening
     - Examples of trade indicators*: KOF Globalization Index; FDI inflows/GDP (%); total trade (imports + exports)/GDP (%); mean applied tariff rate
     - Examples of food/diet indicators: sugar-sweetened beverages imports/sales per capita; consumption per capita for selected food groups (e.g., animal proteins, sugars); average caloric intake; consumption of ‘unhealthy’ foods (% of total spending/caloric intake); supply of caloric sweeteners per capita; ultra-processed products sales per capita
  2. No: stop screening
  - A study monitoring some parts of a trade agreement related to diet but not the actual food environment is excluded
  - A study looking at Canadian trade policy impacts on exports to other countries and consequently the impact on their food environments is excluded

*Additional examples of trade indicators can be found in this article: Cowling K, Thow AM, Pollack Porter K. Analyzing the impacts of global trade and investment on non-communicable diseases and risk factors: a critical review of methodological approaches used in quantitative analyses. Globalization and Health. 2018;14(1):53.
